# Supplementary material for: Predicting water at the protein interface in cryo-EM structures from MD-excess chemical potential
Source: Biophys J. 2026 Jan 27;125(16):4219–32. doi: 10.1016/j.bpj.2026.01.043 (PMC13059003; doi:10.1016/j.bpj.2026.01.043)
Supplement: Supplementary file 1 [file mmc1.pdf]

Biophysical Journal, Volume *125*

Supplemental information

Predicting water at the protein interface in cryo-EM structures from MD-excess chemical potential

Qinfang Sun, Sriram Aiyer, Avik Biswas, Allan Haldane, Sompriya Chatterjee, Nobuyuki Matubayasi, Dmitry Lyumkis, and Ronald M. Levy

Supporting Material

Predicting Water at the Protein Interface in Cryo-EM Structures from MD-Excess Chemical Potential

Qinfang Sun[1,2], Sriram Aiyer[4], Avik Biswas[4,5], Allan Haldane[1,3], Sompriya Chatterjee[1,2,7], Nobuyuki Matubayasi[8], Dmitry Lyumkis[4,6*], Ronald M Levy[1,2,3*]

[1]Center for Biophysics and Computational Biology, Temple University, Philadelphia, PA, 19122;

[2]Department of Chemistry, Temple University, Philadelphia, PA, 19122;

[3]Department of Physics, Temple University, Philadelphia, PA, 19122;

[4]The Salk Institute for Biological Studies, La Jolla, CA, 92037;

[5]Department of Physics, University of California San Diego, La Jolla, CA, 92037;

[6]Graduate School of Biological Sciences, Department of Molecular Biology, University of California San Diego, La Jolla, CA, 92037;

[7]Material Science Institute, University of Oregon, Eugene, OR, 97403;

[8]Division of Chemical Engineering, Graduate School of Engineering Science, Osaka University, Toyonaka, Osaka 560-8531, Japan

*To whom correspondence should be addressed

*dlyumkis@salk.edu

*ronlevy@temple.edu

Table S1 Summary information of 9 cryo-EM structures of apoferritin (Group A) with resolution higher than 1.5 Å and 17 cryo-EM structures of apoferritin (Group B) with resolution between 1.5 and 2.0 Å.

PDB ID	Year of Deposition	Resolution (Å)	Refinement Software	Number of Cryo-EM Water per Chain
**Group A (9)**				
8RQB	2024	1.09	Unknown	110
7A6A	2020	1.15	Refmac	194
8J5A	2023	1.19	Refmac	145
7A4M	2020	1.22	Refmac	110
6Z6U	2020	1.25	Refmac	139
7RRP	2021	1.27	Phenix	102
7A6B	2020	1.33	Refmac	162
7K3V	2020	1.34	Phenix	111
7K3W	2020	1.36	Phenix	111
**Group B (17)**				
6Z9E	2020	1.55	Refmac	146
6Z9F	2020	1.56	Refmac	172
7R5O	2022	1.58	Refmac	139
7KOD	2020	1.65	Phenix	73
8EMQ	2022	1.66	Phenix	124
8EN7	2022	1.68	Phenix	106
6V21	2019	1.75	Phenix	75
8CPU	2023	1.76	Phenix	73
8CPV	2023	1.76	Phenix	71
8CPX	2023	1.76	Phenix	70
7ZG7	2022	1.77	Phenix	111
8CPT	2023	1.79	Phenix	79
8CPW	2023	1.79	Phenix	78
8CPM	2023	1.81	Phenix	81
8CPS	2023	1.82	Phenix	84
6S61	2019	1.84	Phenix	160
7VD8	2021	1.96	Phenix	103

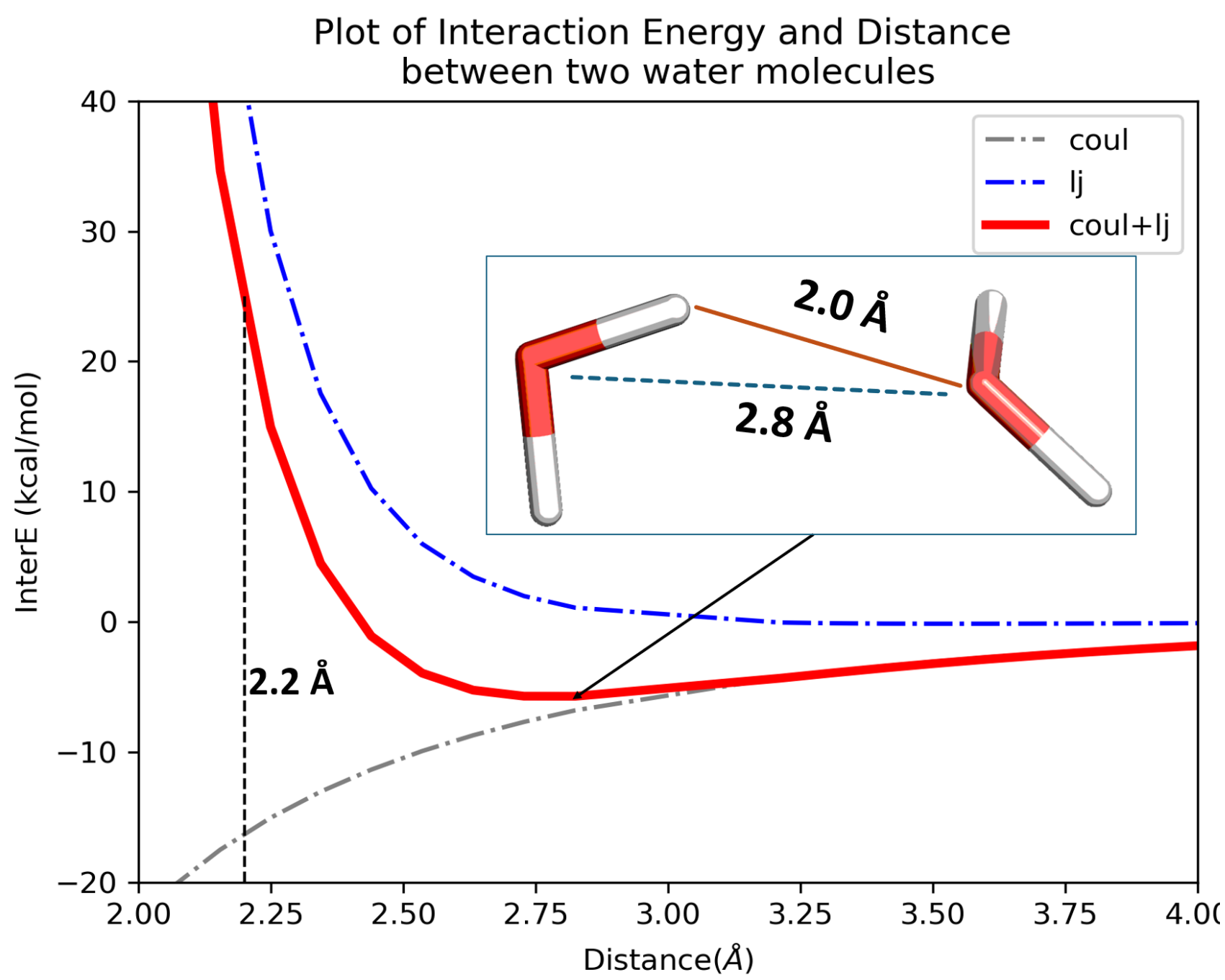

Figure S2: The interaction energy is plotted against the distance for two hydrogen-bonded water molecules (shown in inset) in gas phase using TIP3P model. The curves for Coulombic energy, Lennard-Jones energy and the total interaction energy are shown in grey, blue and red respectively.

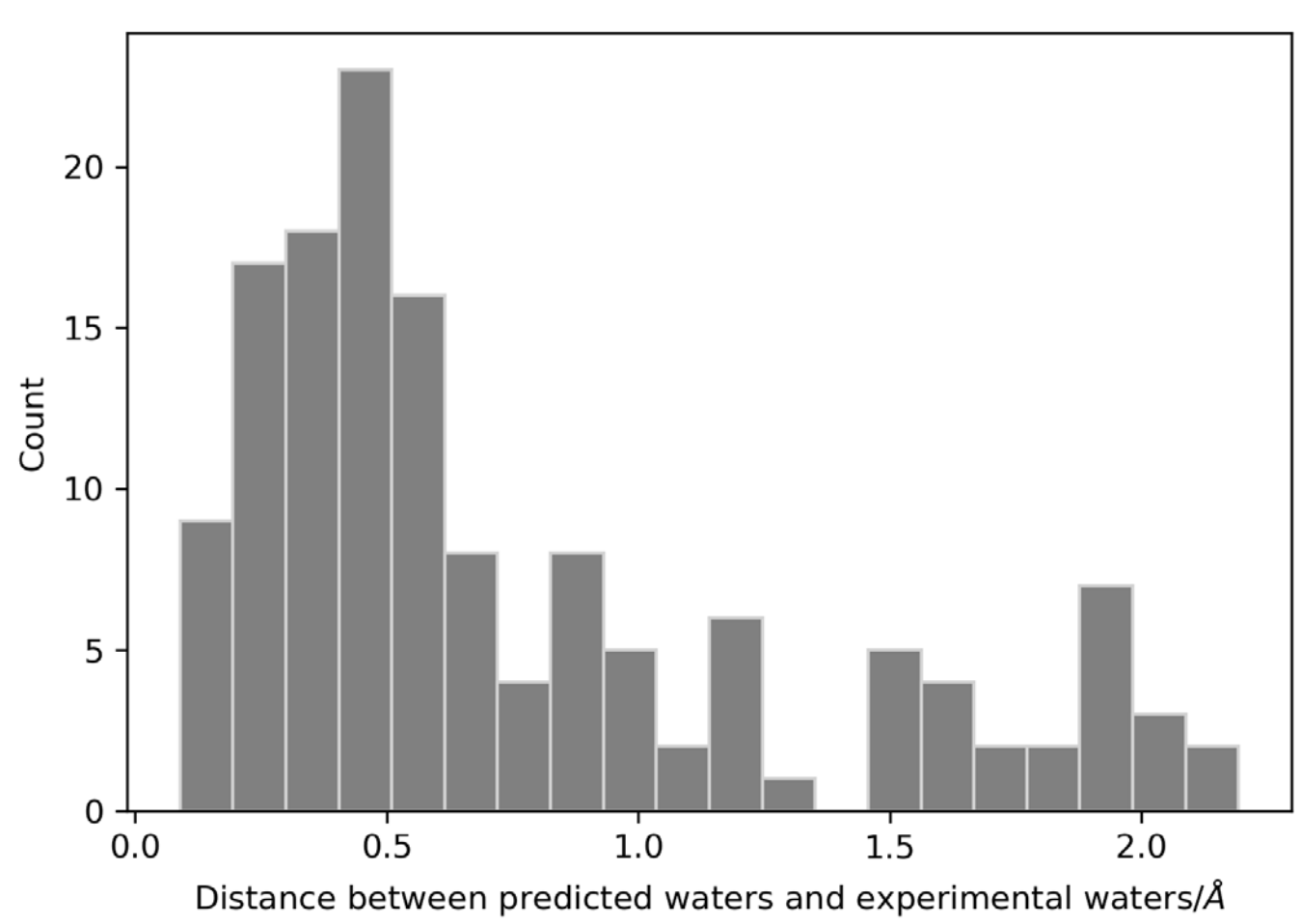

Figure S1: The histogram illustrates the distribution of distances between predicted waters and experimental waters with a 2.2 Å distance cutoff.

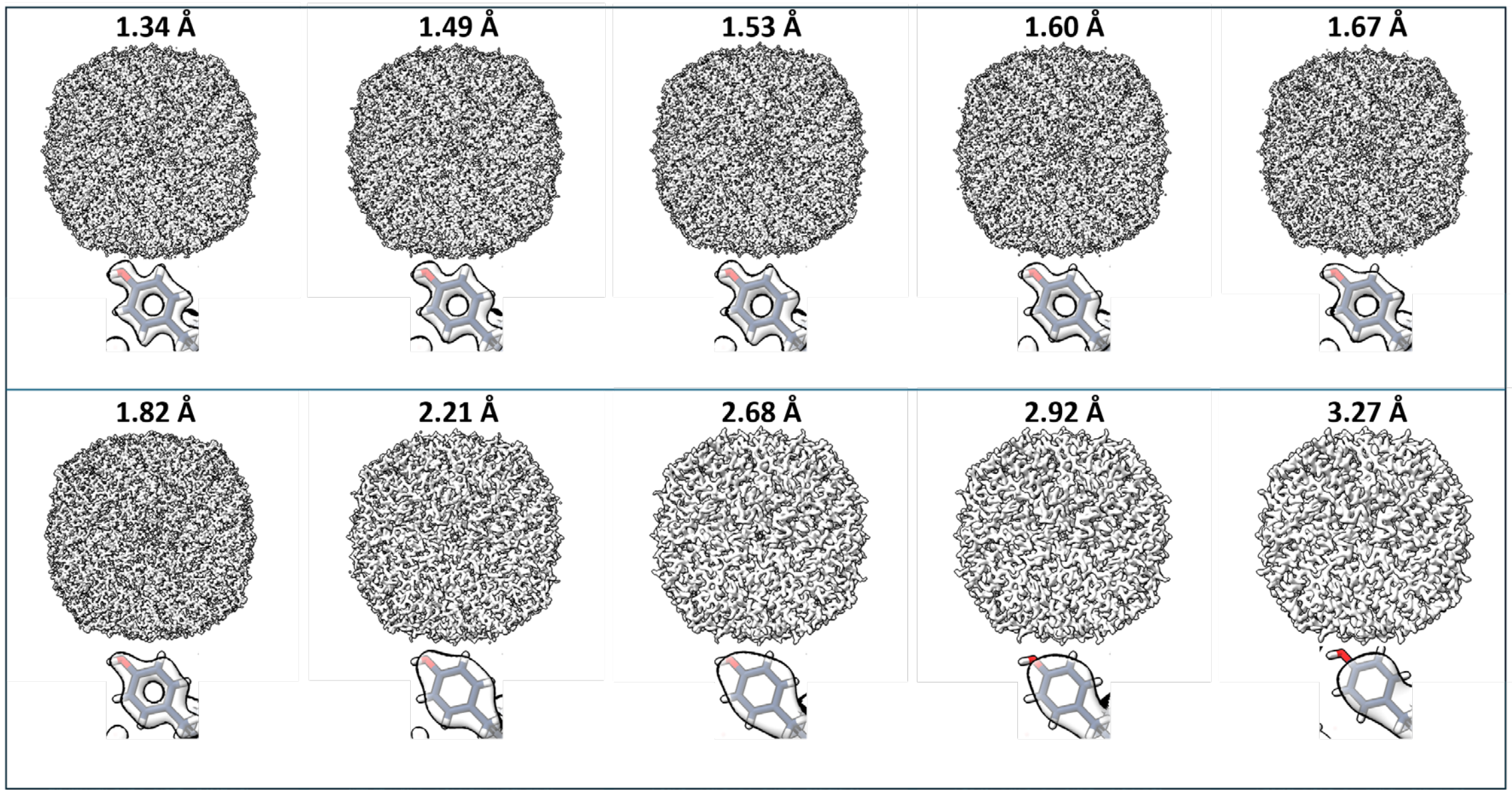

Figure S3: Final apoferritin confidence maps are shown at different resolutions (1.34 Å, 1.49 Å, ... 3.84 Å), with Y168 displayed at the bottom of each respective confidence map visualized at a 1% FDR threshold.

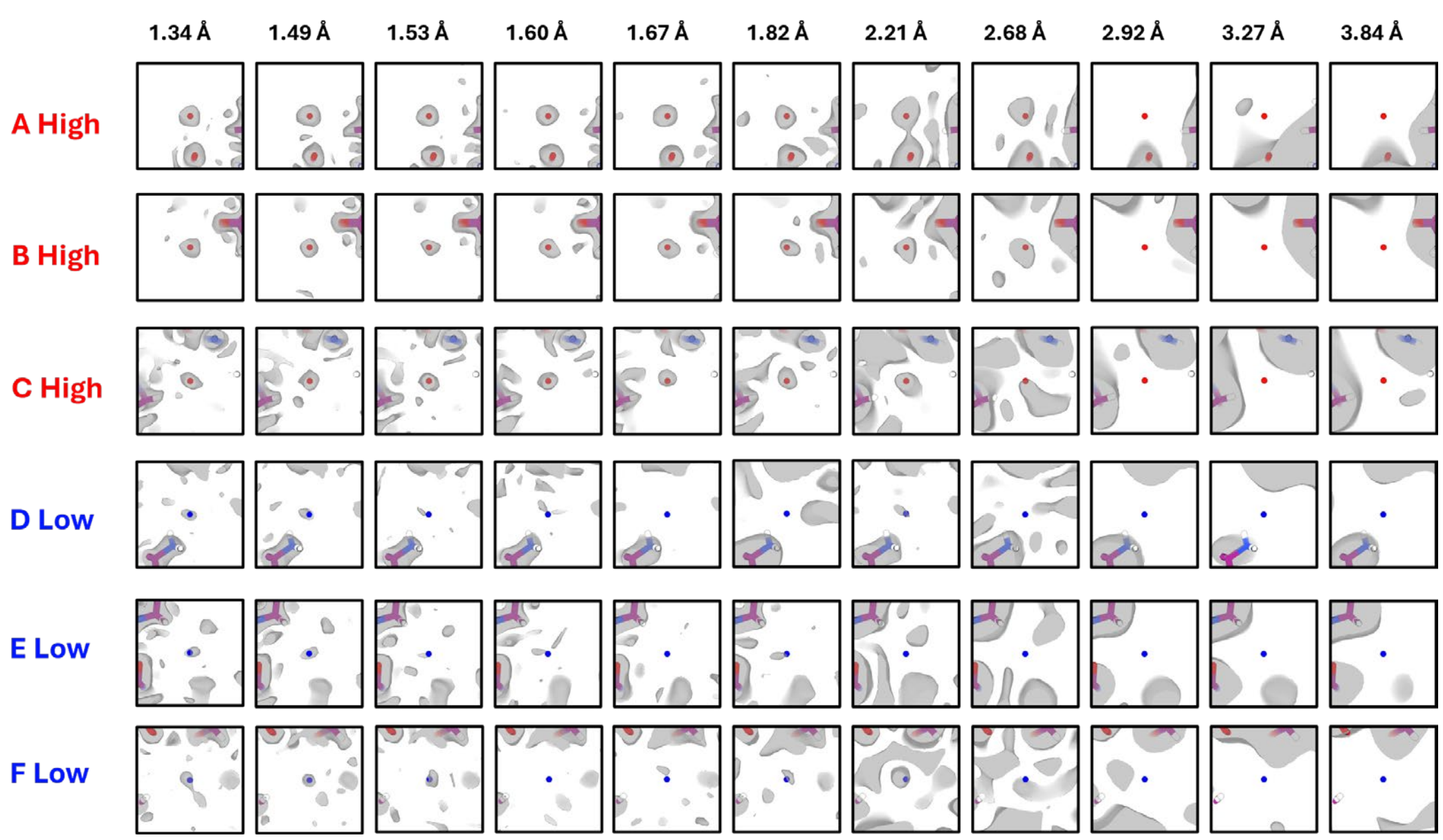

Figure S4: High-consensus (A–C) and low-consensus (D–F) waters are observed in confidence maps derived from cryo-EM data across a range of resolutions (1.34 Å, 1.49 Å, ... 3.84 Å) with a slightly expanded view that shows the surrounding residues. These maps are displayed at a 1% false discovery rate (FDR) threshold.

Table S2 The observation of high- and low-consensus waters in cryo-EM maps across various resolutions (1.34 Å, 1.49 Å, ... 3.84 Å) and at three different false discovery rate (FDR) thresholds: 0.1%, 1%, and 5%. At the different viewing thresholds, 'no' refers to no visible density, 'yes' refers to near-spherical density, 'partial' refers to near-spherical density with minor deviation or partial overlap from centroid position of water in the model, 'noisy' refers to density merging with density from neighboring atoms.

Water Confidence	Resolution (Å)									
	1.34	1.49	1.53	1.6	1.67	1.82	2.21	2.68	2.92	3.84
**lower**	No	No	No at 0.1%, partial overlap at 1 and 5%	No at 0.1 and 1%, partial at 5%	No at 0.1%, partial overlap at 1 and 5%	No	No	No	No	No
**lower**	No at 0.1 and 1%, partial overlap at 5%	No at 0.1%, partial overlap at 1 and 5%	No	No	No	No	Noisy and partial at 0.1%, noisy at 1 and 5%	Noisy and partial	No	No
**lower**	Yes	Partial	Partial at 0.1%, complet e at 1 and 5%	No at 0.1%, partial at 1%, complet e overlap at 5%	Partial	No at 0.1%, partial overlap at 1 and 5%	No	Partial at 0.1%, noisy at 1 and 5%	Noisy and partial at 0.1%, noisy at 1 and 5%	No
**lower**	No at 0.1%, partial at 1 and 5%	No at 0.1 and 1%, partial overlap at 5%	No	No at 0.1%, partial at 1 and 5%	No	No at 0.1%, partial overlap at 1 and 5%	No at 0.1 and 1%, partial overlap at 5%	No	No	No
**lower**	No	No	No	No	No	No	No	No	No	No
**lower**	Partial	Partial	No at 0.1%, partial overlap at 1 and 5%	No at 0.1%, partial at 1 and 5%	No at 0.1%, partial overlap at 1 and 5%	No at 0.1 and 1%, partial overlap at 5%	No at 0.1%, partial and noisy at 1 and 5%	No	No	No
**lower**	No at 0.1 and 1%, partial overlap at 5%	No at 0.1%, partial overlap at 1 and 5%	No	No at 0.1%, partial at 1 and 5%	No at 0.1 and 1%, partial overlap at 5%	Partial	No at 0.1%, partial overlap at 1 and 5%	No	No	No
**lower**	No at 0.1%, partial at 1 and 5%	No	No	No	No	No at 0.1%, partial overlap at 1 and 5%	Noisy and partial at 0.1%, noisy at 1 and 5%	Noisy and partial at 0.1 and 1%, noisy at 5%	No	No
**lower**	Partial	Partial	No at 0.1%, partial overlap at 1 and 5%	No at 0.1 and 1%, noisy at 5%	No	No at 0.1%, partial overlap at 1 and 5%	No	No	No	No
**lower**	Partial	Partial	No at 0.1 and	No at 0.1 and	No	No at 0.1%,	No	No	No	No

			1%, partial at 5%	1, noisy at 5%		partial overlap at 1 and 5%				
**lower**	Yes	Partial	Yes	Yes	Yes	Yes	Yes	Noisy	No at 0.1% and 1% and partial overlap at 5%	No
**lower**	Yes	Yes	Yes	Yes	Yes	Yes	Noisy	Noisy	No	No
**lower**	Yes	Yes	Yes	No at 0.1, noisy at 1 and 5%	Yes	Yes	Yes	No at 0.1, noisy and partial overlap at 1% and 5%	No	No
**lower**	Noisy	Noisy	Yes	Yes	Yes	Yes	Noisy	Noisy and partial	No	No
**lower**	Yes	Yes	Noisy	Yes	Yes	Partial at 0.1%, complete at 1 and 5%	Yes	Yes	Yes	No
**lower**	Yes	Partial at 0.1 and 1%, complete overlap at 5%	No at 0.1%, partial overlap at 1 and 5%	Yes	No at 0.1%, partial overlap at 1 and 5%	Partial	Yes	Partial at 0.1 and 1%, noisy at 5%	No	No
**lower**	Partial at 0.1%, complete overlap at 1 and 5%	Yes	Partial at 0.1 and 1, complete overlap at 5%	Partial at 0.1 and 1%, noisy at 5%	Partial at 0.1, complete overlap at 1 and 5%	Yes	Yes	No at 0.1%, partial at 1 and 5%	No	No
**lower**	Partial at 0.1%, complete overlap at 1 and 5%	Partial	Partial	No	No	No	No at 0.1%, partial and noisy at 1 and 5%	Noisy	No	No
**lower**	Yes	Yes and elongated density	No at 0.1%, overlap at 1 and 5% with elongated density	Yes	No at 0.1%, partial overlap at 1 and 5%	Partial	Noisy	Partial at 0.1 and 1%, partial and noisy at 5%	No	No
**lower**	Partial at 0.1 and 1%, complete overlap at 5%	Yes	Partial at 0.1 and 1, complete overlap at 5%	No	No	No at 0.1%, partial overlap at 1 and 5%	Noisy	Noisy	No	No
**lower**	Yes	Yes	Partial at 0.1, complete overlap	Yes	Partial at 0.1, complete overlap	Yes	Noisy	No	No	No

			at 1 and 5%		at 1 and 5%					
**lower**	Yes	Yes	Yes	Partial	Partial overlap at 0.1%, complete overlap 1 and 5%	Yes	Yes	No	No	No
**lower**	Yes	Partial at 0.1%, complete overlap at 1 and 5%	Yes	Partial at 0.1%, complete overlap at 1 and 5%	Partial	No at 0.1 and 1%, partial overlap at 5%	Noisy	Noisy	No	No
**lower**	Partial at 0.1%, noisy and partial at 1%, noisy at 5%	Yes	Yes	No	No	No at 0.1%, partial overlap at 1 and 5%	Noisy	No at 0.1%, noisy and partial at 1 and 5%	No	No
**high**	Yes	Yes	Yes	Yes	Yes	Yes	Yes	Noisy	No	No
**high**	Yes	Yes	Yes	Yes	Yes	Partial overlap at 0.1 and 1%, complete overlap at 5%	Noisy	Yes	No	No
**high**	Yes	Yes	Yes	Yes	Yes	Yes	Noisy	Yes	No	No
**high**	Yes	Yes	Yes	Yes	Yes	Yes	Yes	Noisy	Partial at 0.1%, noisy at 1 and 5%	No
**high**	Yes	Yes	Yes	Yes	Yes	Yes	Noisy	Partial at 0.1, noisy at 1 and 5%	No at 0.1%, noisy at 1 and 5%	No
**high**	Yes	Yes	Yes	Yes	Partial overlap at 0.1 and 1%, complete overlap at 5%	Partial overlap at 0.1 and 1%, complete overlap at 5%	Noisy	Noisy	No	No
**high**	Yes	Yes	Yes	Yes	Yes	Yes	Yes	Noisy	No at 0.1%, partial overlap at 1% and 5%	No
**high**	Yes	Yes	Yes	Yes	Partial at 0.1, complete overlap at 1 and 5%	Yes	Noisy	Partial at 0.1, complete overlap at 1, noisy at 5%	No	No

**high**	Yes	Yes	Yes	Yes	Yes	Yes	Yes	Partial at 0.1, complet e overlap at 1 and 5%	No	No
**high**	Yes	Yes	Yes	Yes	Yes	Yes	Yes	Noisy	No at 0.1%, partial overlap at 1% and 5%	No
**high**	Yes	Yes	Yes	Yes	Yes	Yes	Yes	Noisy	No	No
**high**	Yes	Yes	Yes	Yes	Noisy	Noisy	No	No at 0.1, noisy and partial overlap at 1% and 5%	No at 0.1%, noisy at 1 and 5%	No
**high**	Yes	Yes	Yes	Yes	Yes	Yes	Yes	Noisy	No	No
**high**	Yes	Yes	Yes	Yes	Yes	Yes	Noisy	Noisy	Noisy	No
**high**	Noisy	Yes	Yes	Yes	Yes	Yes	Noisy	Noisy and partial at 0.1 and 1%, noisy at 5%	No	No
**high**	Yes	Yes	Yes	Yes	Yes	Yes	Yes	Partial at 0.1 and 1%, noisy at 5%	No	No
**high**	Yes	Yes	Yes	Yes	Yes	Yes	Noisy	Noisy	No	No
**high**	Yes	Yes	Yes	Yes	Yes	Yes	Noisy	Noisy and partial at 0.1 and 1%, noisy at 5%	No	No
**high**	Yes	Yes	Yes	Yes	Yes	Yes	Noisy	Partial at 0.1%, complet e overlap at 1 and 5%	No at 0.1%, partial overlap at 1% and 5%	No
**high**	Yes	Yes	Yes	Yes	Yes	Yes	Noisy	Noisy	No	No
**high**	Yes	Yes	Yes	Yes	Yes	Yes	Noisy	Noisy	No at 0.1 and 1%, noisy at 5%	No
**high**	Yes	Yes	Yes	Yes	Yes	Yes	Yes	Yes	No	No
**high**	Yes	Yes	Yes	Yes	Yes	Yes	Yes	No at 0.1, noisy and partial overlap at 1% and 5%	No at 0.1, noisy and partial overlap at 1% and 5%	No

**high**	Yes	Yes	Yes	Yes	Yes	Yes	Noisy	Yes	No at 0.1%, partial at 1 and 5%	No
**high**	Yes	Yes	Yes	Yes	Yes	Yes	Noisy	Noisy	Noisy	No
**high**	Yes	Yes	Yes	Yes	Yes	Yes	Noisy	Yes	Partial at 0.1, complet e overlap at 1 and 5%	No
**high**	Yes	Yes	Yes	Yes	Yes	Yes	Yes	Yes	No at 0.1%, partial overlap at 1% and noisy at 5%	No
**high**	Yes	Yes	Yes	Yes	Yes	Yes	Noisy	Noisy	No at 0.1 and 1%, noisy at 5%	No
**high**	Yes	Yes	Yes	Yes	Yes	Yes	Noisy	Yes	Noisy	No
**high**	Yes	Yes	Yes	Yes	Yes	Yes	Noisy	Noisy	No	No
**high**	Yes	Yes	Yes	Yes	Yes	Yes	Noisy	Noisy	No at 0.1%, partial at 1 and 5%	No
**high**	Yes	Yes	Yes	Yes	Yes	Yes	Noisy	Noisy	No	No
**high**	Yes	Yes	Yes	Yes	Yes	Yes	Noisy	Noisy	No	No
**high**	Yes	Yes	Yes	Yes	Yes	Yes	Yes	Yes	No	No
**high**	Yes	Yes	Yes	Yes	Yes	Yes	Noisy	Noisy	No at 0.1% and 1%, noisy and partial overlap at 5%	No
**high**	Yes	Yes	Yes	Yes	Yes	Yes	Noisy	Yes	No	No
**high**	Yes	Yes	Yes	Yes	Yes	Yes	Yes	Partial	No	No
**high**	Yes	Yes	Yes	Yes	Yes	Yes	Yes	Noisy	No	No
**high**	Yes	Yes	Yes	Yes	Yes	Yes	Yes	No at 0.1, noisy and partial overlap at 1% and 5%	No	No
**high**	Yes	Yes	Yes	Yes	Yes	Yes	Noisy	Partial at 0.1%, noisy at 1 and 5%	No	No
**high**	Yes	Yes	Yes	Yes	Yes	Yes	Noisy	Noisy	No	No
**high**	Yes	Yes	Yes	Yes	Yes	Partial at 0.1 and	Yes	Noisy and	No	No

						1%, complete at 5%		partial at 0.1%, noisy at 1 and 5%		
**high**	Yes	Yes	Yes	Yes	Yes	Yes	Yes	Noisy	Yes	No
**high**	Yes	Yes	Yes	Yes	Yes	Noisy	Noisy	Noisy	No	No
**high**	Yes	Yes	Yes	Yes	Yes	Yes	Noisy	Noisy	No	No
**high**	Yes	Yes	Yes	Yes	Yes	Yes	Noisy	Yes	No	No
**high**	Yes	Yes	Yes	Yes	Yes	Partial at 0.1 and 1%, complete at 5%	Noisy	Yes	No	No
**high**	Yes	Yes	Yes	Yes	Yes	Yes	Noisy	Partial at 0.1%, complete overlap at 1 and 5%	No	No
**high**	Yes	Yes	Yes	Yes	Yes	Yes	Noisy	Noisy	Partial and noisy	No
**high**	Yes	Yes	Yes	Yes	Yes	Yes	Noisy	Partial at 0.1 and 1%, complete overlap at 5%	No	No
**high**	Yes	Yes	Yes	Yes	Yes	Yes	Yes	Noisy	No at 0.1%, partial at 1% and complete at 5%	No
**high**	Yes	Yes	Yes	Yes	Yes	Yes	Yes	Yes	No	No
**high**	Yes	Yes	Yes	Yes	Yes	Yes	Noisy	Yes	Noisy	No

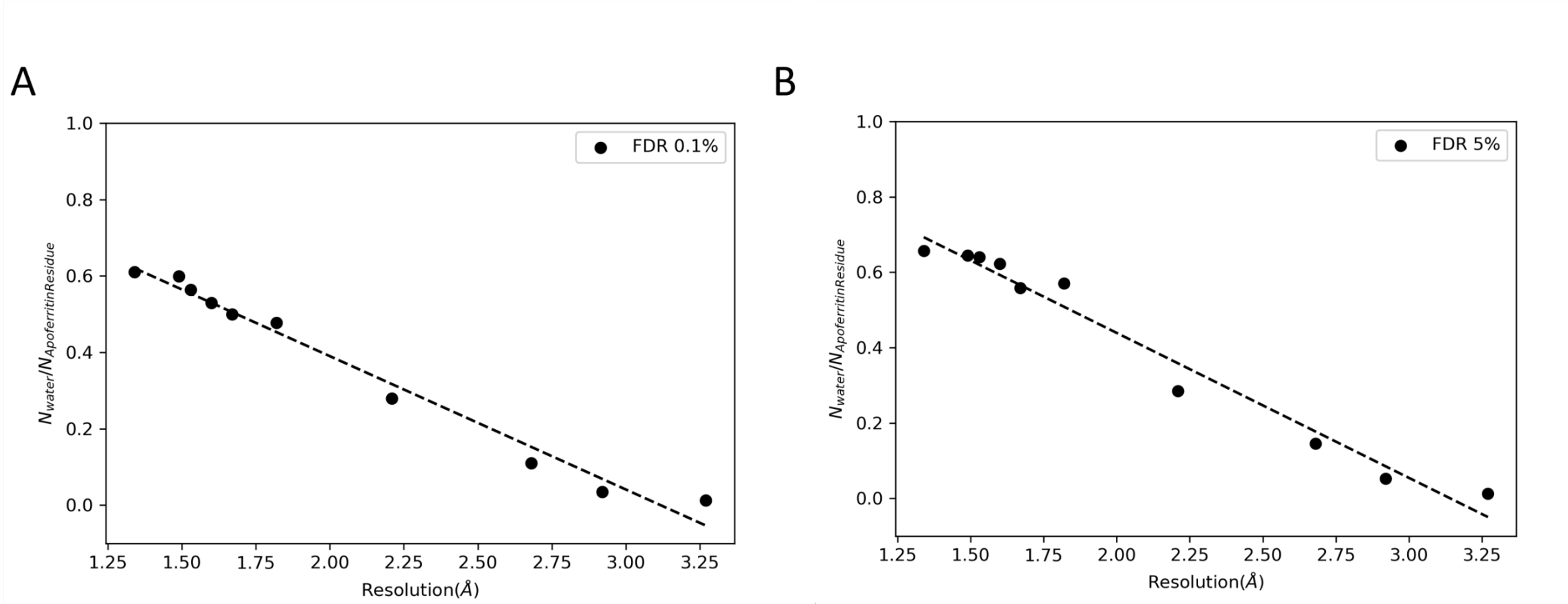

Figure S5: The plots showing the number of water molecules per apoferritin residue as a function of map resolution are based on data analyzed on confidence maps at 0.1% (**A**) and 5% (**B**) false discovery rate (FDR) thresholds.

Table S3 Comparison of water molecule rankings. (A) The top 60 water molecules ranked by their calculated interaction energies in Figure 3A. (B) The top 60 water molecules ranked by their WT values in Figure 3B. WT values were determined based on the experimentally resolved water positions. The interaction energy for each water molecule was computed with respect to the surrounding protein environment. Waters highlighted in green denote high-consensus sites, whereas waters highlighted in cyan indicate those that are present in both Table A and Table B.

A

WT_rank_ID	IE_rank_ID	cluster type	WT/kcal/mol	interEwo_Zn/kcal/mol	indirect part/kcal/mol
1	1	5	-4.72	-23.18	18.47
184	2	6	-1.01	-22.03	21.02
162	3	1	-2.67	-20.63	17.97
185	4	2	-1.00	-16.93	15.93
50	5	4	-4.19	-16.05	11.86
93	6	4	-3.65	-15.28	11.63
131	7	6	-3.13	-15.20	12.07
42	8	8	-4.27	-15.04	10.77
26	9	1	-4.43	-14.94	10.52

22	10	5	-4.44	-14.79	10.34
23	11	8	-4.44	-14.75	10.31
60	12	2	-4.05	-14.75	10.70
100	13	5	-3.51	-14.37	10.86
31	14	8	-4.38	-14.37	9.99
81	15	7	-3.82	-14.27	10.45
49	16	9	-4.19	-14.11	9.91
168	17	5	-2.53	-13.84	11.31
24	18	9	-4.44	-13.58	9.13
7	19	9	-4.60	-13.42	8.82
147	20	4	-2.92	-13.26	10.34
39	21	9	-4.31	-13.19	8.88
6	22	6	-4.61	-13.14	8.53
55	23	6	-4.15	-13.06	8.91
8	24	8	-4.59	-12.82	8.23
10	25	6	-4.57	-12.80	8.23
91	26	9	-3.69	-12.78	9.09
107	27	2	-3.43	-12.73	9.30
17	28	9	-4.51	-12.71	8.20
178	29	1	-1.67	-12.67	11.00
36	30	3	-4.35	-12.65	8.30
35	31	9	-4.35	-12.53	8.18
80	32	3	-3.82	-12.42	8.60
3	33	7	-4.66	-12.37	7.71
181	34	9	-1.46	-11.99	10.53
71	35	3	-3.93	-11.85	7.92
97	36	1	-3.54	-11.81	8.27

95	37	9	-3.62	-11.72	8.10
116	38	6	-3.34	-11.52	8.18
160	39	8	-2.67	-11.51	8.84
117	40	3	-3.33	-11.47	8.13
174	41	3	-2.20	-11.41	9.21
78	42	3	-3.85	-11.38	7.52
54	43	9	-4.16	-11.31	7.16
47	44	9	-4.20	-11.20	7.00
34	45	7	-4.35	-11.05	6.70
186	46	1	-1.00	-11.04	10.04
20	47	9	-4.49	-10.89	6.40
105	48	2	-3.47	-10.62	7.15
123	49	3	-3.24	-10.60	7.36
27	50	9	-4.41	-10.60	6.19
115	51	6	-3.34	-10.12	6.78
29	52	9	-4.39	-10.05	5.66
101	53	1	-3.51	-10.00	6.49
58	54	9	-4.09	-9.95	5.86
121	55	1	-3.25	-9.81	6.56
5	56	5	-4.62	-9.77	5.15
125	57	9	-3.19	-9.74	6.55
171	58	7	-2.43	-9.64	7.21
76	59	6	-3.87	-9.53	5.67
111	60	5	-3.40	-9.37	5.98
	ave	5.63 ± 2.85	-3.64 ± 0.96	-12.84 ± 2.78	9.2 ± 3.04

B

WT_rank_ID	IE_rank_ID	cluster type	WT/kcal/mol	interEwo_Zn/kcal/mol	indirect part/kcal/mol
1	1	5	-4.72	-23.18	18.47
2	141	9	-4.70	-5.35	0.66
3	33	7	-4.66	-12.37	7.71
4	71	6	-4.65	-8.36	3.71
5	56	5	-4.62	-9.77	5.15
6	22	6	-4.61	-13.14	8.53
7	19	9	-4.60	-13.42	8.82
8	24	8	-4.59	-12.82	8.23
9	86	7	-4.58	-7.30	2.71
10	25	6	-4.57	-12.80	8.23
11	104	8	-4.56	-6.62	2.06
12	91	9	-4.55	-7.16	2.61
13	132	8	-4.55	-5.64	1.09
14	153	7	-4.54	-4.84	0.30
15	62	8	-4.53	-9.32	4.79
16	168	9	-4.52	-3.94	-0.57
17	28	9	-4.51	-12.71	8.20
18	68	9	-4.51	-8.68	4.17
19	136	9	-4.50	-5.58	1.08
20	47	9	-4.49	-10.89	6.40
21	76	9	-4.46	-8.02	3.56
22	10	5	-4.44	-14.79	10.34

23	11	8	-4.44	-14.75	10.31
24	18	9	-4.44	-13.58	9.13
26	9	1	-4.43	-14.94	10.52
25	98	9	-4.43	-6.84	2.41
27	50	9	-4.41	-10.60	6.19
28	142	3	-4.40	-5.35	0.95
29	52	9	-4.39	-10.05	5.66
30	124	6	-4.38	-5.90	1.52
31	14	8	-4.38	-14.37	9.99
32	128	9	-4.36	-5.81	1.45
33	94	9	-4.36	-7.02	2.66
35	31	9	-4.35	-12.53	8.18
34	45	7	-4.35	-11.05	6.70
36	30	3	-4.35	-12.65	8.30
37	118	9	-4.32	-6.27	1.95
38	152	9	-4.32	-4.86	0.55
39	21	9	-4.31	-13.19	8.88
41	61	9	-4.28	-9.36	5.09
40	160	3	-4.28	-4.43	0.16
42	8	8	-4.27	-15.04	10.77
43	150	9	-4.24	-5.00	0.76
44	80	9	-4.23	-7.69	3.46
45	66	8	-4.22	-8.94	4.73
46	75	9	-4.20	-8.18	3.97
47	44	9	-4.20	-11.20	7.00
48	180	1	-4.20	-2.00	-2.20
49	16	9	-4.19	-14.11	9.91

50	5	4	-4.19	-16.05	11.86
51	121	8	-4.17	-6.23	2.06
52	138	9	-4.17	-5.50	1.33
53	99	8	-4.17	-6.81	2.65
54	43	9	-4.16	-11.31	7.16
55	23	6	-4.15	-13.06	8.91
56	100	8	-4.15	-6.75	2.60
57	147	9	-4.09	-5.03	0.94
58	54	9	-4.09	-9.95	5.86
59	107	9	-4.05	-6.50	2.45
60	12	2	-4.05	-14.75	10.70
	ave	7.46 ± 2.21	-4.37 ± 0.17	-9.57 ± 4.01	5.19 ± 3.99
