# Supplementary material for: Predicting water at the protein interface in cryo-EM structures from MD-excess chemical potential
Source: Biophys J. 2026 Jan 27;125(16):4219–32. doi: 10.1016/j.bpj.2026.01.043 (PMC13059003; doi:10.1016/j.bpj.2026.01.043)
Supplement: Supplementary file 2 [file mmc2.pdf]

Biophysical Journal
Article

Biophysical Society

Predicting water at the protein interface in cryo-EM structures from MD-excess chemical potential

Qinfang Sun,[1,2] Sriram Aiyer,[4] Avik Biswas,[4,5] Allan Haldane,[1,3] Sompriya Chatterjee,[1,2,7] Nobuyuki Matubayasi,[8] Dmitry Lyumkis,[4,6,*] and Ronald M. Levy[1,2,3,*]

[1]Center for Biophysics and Computational Biology, Temple University, Philadelphia, Pennsylvania; [2]Department of Chemistry, Temple University, Philadelphia, Pennsylvania; [3]Department of Physics, Temple University, Philadelphia, Pennsylvania; [4]The Salk Institute for Biological Studies, La Jolla, California; [5]Department of Physics, University of California, San Diego, La Jolla, California; [6]Graduate School of Biological Sciences, Department of Molecular Biology, University of California, San Diego, La Jolla, California; [7]Material Science Institute, University of Oregon, Eugene, Oregon; and [8]Division of Chemical Engineering, Graduate School of Engineering Science, Osaka University, Toyonaka, Osaka, Japan

ABSTRACT Predicting the positions of water molecules at the protein interface remains a formidable challenge in structural biology, fueling active research in this field. Here, we present a novel approach based on molecular dynamics (MD) simulations that utilize statistical thermodynamic signatures of water at protein interfaces that can be used to improve the accuracy of water placement in maps derived by cryogenic electron microscopy (cryo-EM). We employ an analysis based on the excess chemical potential, or the work to transfer (WT) a water molecule from the bulk to the interface. WT is a measure of the thermodynamic balance between the interaction energy of an interfacial water molecule with the protein and its free energy of interaction with all the other solvent molecules. WT is proportional to the log ratio of the local density of water molecules at the protein interface to the bulk density. Using apoferritin as a benchmark system, we found that 85% of the top 100 water locations with the most favorable excess chemical potential values are observed in one or more structures whose locations were determined from high-resolution cryo-EM maps deposited in the Protein Data Bank (PDB). Seventy percent of the top 200 water locations indexed by excess chemical potential were also observed in PDB structures derived from the cryo-EM maps. The MD simulations are performed without experimental density restraints, yet the water positions with favorable WT values correlate strongly with their corresponding position within experimentally defined maps. This work paves the way for the development of a cryo-EM water placement and refinement tool that integrates MD simulations of the excess chemical potential with cryo-EM data for accurate modeling of water networks.

SIGNIFICANCE Predicting water molecules at protein interfaces is critical for structural biology but remains a major challenge. This study introduces a novel molecular dynamics (MD) approach based on the excess chemical potential—or "work to transfer"—of water. Using apoferritin as a benchmark, we demonstrate that this approach accurately predicts water positions found in high-resolution cryogenic electron microscopy (cryo-EM) maps, without experimental density restraints. With 85% of top-ranked water sites matching experimental data, this work paves the way for advanced cryo-EM refinement tools that integrate MD simulations with experimental data, enabling more accurate and automated modeling of the essential water networks surrounding proteins.

INTRODUCTION

Water solvation plays a key role in protein structural stability and function. Water is critical for ligand pharmacology and can contribute significantly to ligand-binding thermodynamics. Water displacement by a single chemical group of a bound ligand can contribute substantially to gains in binding affinity from both enthalpic and entropic considerations (1–4). Water also plays a critical role in drug resistance. For example, displacement of waters bound within the active site of lentiviral integrases has been proposed to be the initiating step for a key mechanism of drug resistance (5,6). Many such examples exist in the literature (7–10).

Submitted September 10, 2025, and accepted for publication January 23, 2026.

*Correspondence: dlyumkis@salk.edu or ronlevy@temple.edu

Editor: Frauke Graeter.

https://doi.org/10.1016/j.bpj.2026.01.043

© 2026 The Author(s). Published by Elsevier Inc. on behalf of Biophysical Society.
This is an open access article under the CC BY license (http://creativecommons.org/licenses/by/4.0/).

Thus, the proper identification of water molecules is crucial for pharmacological efforts to improve drug design. Despite their significance, accurately placing water molecules within protein structures remains a significant challenge due to technical difficulties in resolving and modeling their positions (11).

Accurate identification of water is still an ongoing area of research even in atomic-resolution X-ray structures and is becoming an important focus in cryogenic electron microscopy (cryo-EM) map analysis (12,13). In X-ray crystallography, anomalous dispersion methods combined with computational techniques for generating omit maps can be used to distinguish between ions and ordered waters, owing to different scattering potentials of these entities. In cryo-EM, omit maps can also be employed for placing water molecules, but their use typically requires resolving the map to sub-2-Å, such that density for hydrogen atoms can be discerned (14). In principle, the ability of the electron beam to differentially scatter oxygen at different spatial frequencies can be exploited to visualize water molecules, but approaches built on these ideas have not yet gained traction (15,16). The typical workflow for modeling waters into cryo-EM maps involves assigning waters into bumps of density (without the use of omit maps and, typically, at user-defined contour values for the map), refining the model against the experimental map, and validating the refined positions using knowledge of chemistry and/or tools such as Undowser in MolProbity (17). This process is iterated until a suitably high-quality model is obtained.

The experimental placement and validation of waters into cryo-EM densities remains prone to error, especially for maps derived at resolution below ≈2.5 Å. Several computer-aided methods have been devised to place water into experimental densities to simplify the above steps and/or to improve and validate the accuracy of placement. Recent advancements involve the use of neural networks to position water molecules around proteins (18–20). The metric ion classification tool is the latest deep-learning method that is derived from training data on published X-ray and cryo-EM structures and facilitates distinguishing between waters and ions (21).

Molecular dynamics (MD)-based approaches have a long history of use as a tool to solvate protein and nucleic acid structures determined by X-ray crystallography and NMR (22–25). In early work using myoglobin as a model system, hydration site consistency with MD was compared across three crystal structures (26), conceptually similar to the approach taken here using apoferritin as a model system. MD methods can provide valuable information to facilitate placing and refining waters in cryo-EM structures by offering a likelihood-based method for placing waters at key locations, validating observed densities in cryo-EM maps, and serving as an alternative when water densities are not fully distinguishable from noise, which is often the case except at the highest resolutions. By simulating the behavior of water molecules around the macromolecule, MD can help identify probable water positions and improve the accuracy of solvent identification and refinement in cryo-EM structures. Empirical techniques based on interaction energies (27) or knowledge-based models have been widely applied to water modeling over the past few decades. However, their reliance on crude approximations—particularly the treatment of the binding energy of water without accounting for entropy—limits both their accuracy and their transferability across different chemical and physical environments (28). Papoian et al. (29) explored the complex role of water in protein folding and function, highlighting the limitations of traditional energy calculations and emphasizing the need for more detailed thermodynamic metrics. Young et al. (30) used MD simulations combined with inhomogeneous fluid solvation theory to evaluate the binding enthalpies and entropies of interfacial water molecules in protein-ligand complexes, demonstrating how the properties of water molecules that solvate confined regions of protein active sites can contribute to enhanced binding affinities. Grand canonical Monte Carlo methods (31) allow water molecules to fluctuate in protein cavities but require complex and time-consuming simulations to approximate realistic conditions (32). Alternatively, free-energy methods such as free-energy perturbation and thermodynamic integration can calculate absolute binding free energies of water molecules (33,34), but the large number of simulations needed makes routine application challenging. Recently, Roh et al. integrated cryo-EM and MD simulations to cross-validate ordered water molecules along the proton path of yeast V_o complex in cryo-EM maps (35). An MD-based framework using peak water density has also been employed to identify and place water molecules into cryo-EM maps of RNA, capturing both the static and semi-ordered hydration layers and providing a more comprehensive view of the hydration landscape(36). However, the predictions exhibited a substantial accuracy gap relative to experimental density and were structurally region dependent (36).

Given the limitations of current techniques, we sought to develop a molecular simulation methodology grounded in statistical thermodynamics that is capable of rapidly and accurately determining the water locations in cryo-EM structures. The statistical thermodynamic characterization of interfacial phenomena is essential for understanding numerous biophysical and chemical events, including protein folding, ion channel gating, membrane permeability, and the formation of protein assemblies. It also has relevance to the energy industry for applications such as electrolyte transport through pores (37–40). There is a large body of literature reporting the use of MD simulations and companion statistical methods to analyze the structural and thermodynamic properties of water at protein interfaces (41–43). According to multiple studies, analyzing the excess chemical potential (WT: the work to transfer a water molecule from the bulk to the interface) is central to the problem

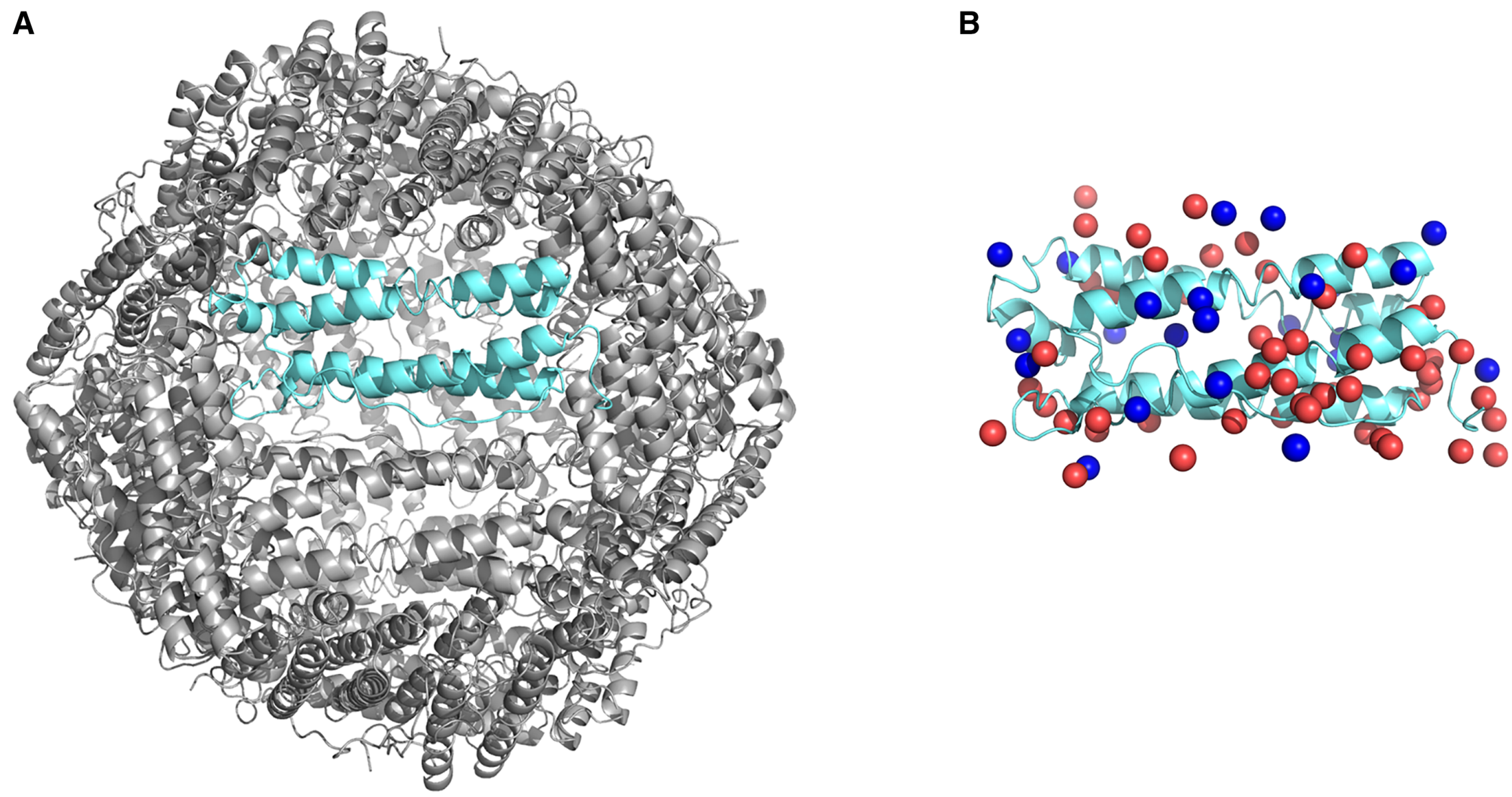

FIGURE 1 The quaternary structure of apoferritin and a close-up view of a single monomer (*cyan*). (*A*) Apoferritin is a protein shell consisting of 24 identical subunits (PDB: 7A4M). (*B*) Selected high-consensus (*red*) and low-consensus (*blue*) waters are shown on a single apoferritin subunit (PDB: 7A4M).

of interpreting interfacial processes and the statistical thermodynamics of liquids (44–46). The excess chemical potential of solvent at the protein interface is expressed as the difference between the free energy of insertion of the solvent at the interface and its insertion in the bulk far from the protein. WT can also be determined by the ratio of the local density of water molecules around a protein to the bulk density. We have recently shown that designing tighter binding ligands can be aided by knowledge of the excess chemical potential of hydrating water at protein-ligand binding sites (4).

Here, we develop a procedure built on the quantitative evaluation of the excess chemical potential to assign waters at the protein interface—without any experimental density restraints—and used these signatures to identify and cross-validate the locations of waters in cryo-EM structures. The target system we employ is apoferritin (shown in Fig. 1 *A*), which plays a role in iron storage and regulation within living organisms (47). Over the last decade, apoferritin has been routinely used as one of the primary benchmark systems for developing novel methods for cryo-EM structure determination. Most of the highest atomic-resolution cryo-EM structures solved have been that of apoferritin (48,49). As an initial benchmarking exercise, we first compare water positions across nine high-resolution (better than 1.5 Å) experimental structures of apoferritin, identifying clusters of waters present in all nine structures. We use these water clusters to develop criteria for assigning water positions as "high consensus" (present in five or more high-resolution structures) or "low consensus" (present between one and four high-resolution structures) based on the number of high-resolution cryo-EM maps that identified water at a given location. Next, we analyze the statistical thermodynamic properties of these high-consensus and low-consensus interfacial water locations by evaluating their interaction energies with apoferritin and excess chemical potentials (WT). This analysis provides insights into the correlation between the strength of the excess chemical potential and the likelihood that a water location was identified in the benchmark set of apoferritin structures with high- or low-consensus water positions. Finally, we evaluate the excess chemical potential of water as a function of position within the first hydration shell at the protein interface and use these signatures to identify the locations of high-density interfacial waters. We then compare the de novo MD-based placement of water molecules with water locations identified in apoferritin cryo-EM structures deposited in the Protein Data Bank (PDB). We investigate potential reasons for inconsistencies in the identification of water positions when comparing the high excess chemical potential sites with water positions listed in a set of apoferritin PDB structures. We speculate why some locations with highly favorable excess chemical potential are not resolved as water density in the cryo-EM maps.

This study contributes to the development of new tools for identifying waters in cryo-EM structures based on the principles of statistical thermodynamics in the absence of experimental density restraints. By understanding the statistical thermodynamic properties of water at the protein interface, researchers can gain deeper insights into how solvation contributes to the stability of folded proteins, the role played by solvent in protein function, and how to design better therapeutic molecules that exploit the unique environment at protein-water interfaces.

MATERIALS AND METHODS

System preparation and MD simulations

High-resolution cryo-EM structures serve as a foundation for identifying water molecules at protein interfaces. The starting structure of apoferritin for the MD simulation is derived from the cryo-EM structure (PDB: 7A4M (chain A) shown in Fig. 1 *B*). The MD simulations were conducted using the GROMACS 2020.3 suite. The ff99SB force field together with the explicit TIP3P solvation model was adopted to describe the protein and the solvent effects. Apoferritin was placed at the center of a cubic box, and the distance between apoferritin and the edge of the box was set to be $\geq$1.0 nm. The final simulation box contains apoferritin, 23,038 randomly added water molecules, and 5 Na^+ ions, with a total of 71,892 atoms in the system. The procedure began with energy minimization to relax the initial configuration, followed by a 100-ps equilibration in the NVT ensemble and a 5-ns equilibration in the NPT ensemble, both with restraints on the protein heavy atoms. The production run was carried out in the NPT ensemble with all protein atoms fixed for 1 μs at a temperature of 300 K and pressure of 1 atm. Temperature control was achieved using a modified Berendsen thermostat, while pressure was regulated to 1 bar with a compressibility of 4.5×10^{-5} bar using the Parrinello-Rahman barostat (50). Electrostatic interactions were treated using the particle mesh Ewald (51) method with a real-space cutoff of 1.0 nm. The MD simulations were executed with a time step of 1 fs, and trajectory files were saved every 0.2 ps. For the WT analysis, 100,000 frames were extracted from the 1-μs trajectory by selecting every fifth frame within the 300- to 400-ns time window. The WT analysis was also repeated using frames from 800 to 900 ns, and the results were consistent with those from the first interval.

Interaction energy calculations for high- and low-consensus waters

The methodology for categorizing high- and low-consensus water positions (196 water clusters shown in Fig. 3) is detailed in the results section. We first incorporated 196 water molecules, one representative of each cluster, into a single apoferritin monomer (7A4M(52)), solvated the system using the TIP3P water model in a cubic box, and neutralized by adding 5 Na^+ ions, as described in "system preparation and MD simulations." The process began with energy minimization to relax the initial configuration, followed by a 100-ps equilibration in the NVT ensemble and a subsequent 100-ps equilibration in the NPT ensemble. During the minimization step, the 196 water oxygens were harmonically restrained, while both the 196 water oxygens and protein heavy atoms were restrained during the NVT and NPT equilibration simulations. The rest of the parameters are the same as described in "system preparation and MD simulations." Lastly, the total interaction energy between the protein and each water was calculated using the final frame of the NPT equilibration and extracted by the gmx_energy module. Nonbonded interaction energies (Coulombic and Lennard-Jones) were computed to quantify the strength of interactions between the protein and each water.

WT calculations for high- and low-consensus waters

The WT for the 196 unique water positions in Fig. 3 *B* is calculated as described here. In Fig. 2 and Eq. 1, the excess chemical potential of a water molecule can be estimated as the free-energy difference $\Delta F(\mathbf{x}) - \Delta F(\infty)$ or the density ratio $-kT \ln\frac{\rho(\mathbf{x})}{\rho(\infty)}$ ($\rho(\infty) = 0.033 Å^{-3}$).

$$\Delta F(\mathbf{x}) - \Delta F(\infty) = \mathrm{WT}(\mathbf{x}) \cong -kT \ln\frac{\rho(\mathbf{x})}{\rho(\infty)} = E_{\mathrm{p-w}}(\mathbf{x}) + \omega(\mathbf{x}). \quad (1)$$

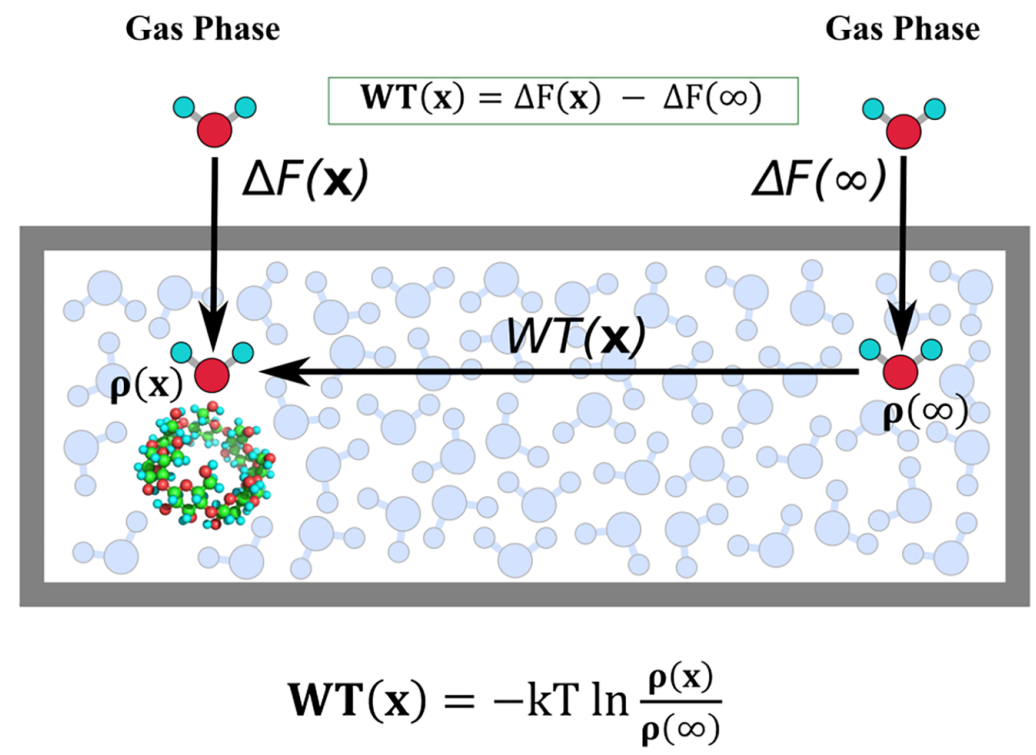

FIGURE 2 The excess chemical potential of a water molecule at **x** can be estimated by the difference between the solvation free energy for growing a water molecule at **x**, $\Delta F(\mathbf{x})$, and the solvation free energy for growing a water molecule in the bulk $\Delta F(\infty)$ (or the pure liquid $\Delta F(0)$). The excess chemical potential can also be calculated by the log ratio of the local density (ρ(x)) of water molecules around a protein to the bulk density ($\rho(\infty)$: $0.033Å^{-3}$).

$E_{\mathrm{p-w}}(\mathbf{x})$, the direct term, is the average interaction energy of the protein with a water at **x**, and $\omega(\mathbf{x})$, the indirect term, is the contribution to the free-energy difference between a water at **x** and a water in the bulk, due to its interactions with all of the other solvent species, water, and ions in the system, interacting with the water at **x** relative to the corresponding interactions in the bulk (4,44,52–54). Numerically, we use $-kT \ln\frac{\rho(\mathbf{x})}{\rho(\infty)}$ to estimate WT (dV) by gridding space into very small cubic voxels used to calculate the density $\boldsymbol{\rho}(\mathbf{x})$. For calculating WT for the 196 water positions identified in the PDB structures, we created a grid around each reference oxygen position, extending 0.5 Å in the x, y, and z directions. The grid consists of fine cubic voxels with an edge length of $a = 0.005$ Å and volume $dV = a^3$. Subsequently, we selectively compute WT using the densest fine voxels, which is the top 1% of the 8 million cubic voxels for each reference position, or $n = 80{,}000$ fine voxels. The Boltzmann average WT is calculated as the sum of the exponential of all WT (dV) values (Eq. 1) divided by the sum of volume of the n fine voxels (Eq. 2).

$$\left\langle e^{-\beta \mathrm{WT}(V)} \right\rangle = \frac{\int_0^R e^{-\beta \mathrm{WT}(dV)} dV}{\int_0^R dV} \rightarrow \mathrm{WT}(V) \quad (2)$$
$$= -RT \times \ln\frac{\sum e^{-\beta \mathrm{WT}(dV)} dV}{V}$$
$$= -\mathrm{RT} \times \ln\frac{\sum e^{-\beta \mathrm{WT}(dV)}}{n}.$$

Waters predicted by excess chemical potential

We first search for locations of high water density by counting all water molecules within 3.25 Å of apoferritin (55), covering the first hydration shell, from 100,000 frames of MD simulations (see "system preparation and MD simulations") using a coarser grid with voxel edge length of 0.1 Å, resulting in a total of approximately 240 million coarse voxels. This grid is also used to visualize the water distribution around apoferritin. A sliding cube of $10 \times 10 \times 10$ such voxels, with a total edge length of 1 Å, is used as a convolution window to identify the 1-Å-sized regions with the most water molecules. We then filter these so that each 1-Å cube must be at least 2.2 Å apart from each other and at least 2.25 Å away from apoferritin.

From 100,000 frames of MD trajectory, the average position of the waters within each qualifying 1-Å cube is calculated and set as the predicted water position. The center of each qualifying 1-Å cube is used as the reference position for the subsequent WT calculations.

Evaluation metrics: Precision and recall

$$\text{recall} = \frac{\text{TP}}{\text{TP}+\text{FN}} \qquad \text{precision} = \frac{\text{TP}}{\text{TP}+\text{FP}}. \tag{3}$$

True-positive (TP) predictions refer to the number of predicted water molecules that match experimental water positions in apoferritin within 2.2 Å. False-positive (FP) predictions represent the number of the predicted water molecules that do not match any experimental water, while false-negative (FN) predictions indicate the number of experimental water molecules that are not matched to any predicted water (Eq. 3). In this evaluation, each predicted water is assigned to its closest experimental water, with only one prediction counted per experimental water.

Cryo-EM image processing by confidence maps

We obtained raw movie frames from Electron Microscopy Public Image Archive (EMPIAR) deposition 10424 and used 1689 movies for the entire analysis. All the data-processing steps were carried out in CryoSparc v.4.6 (56,57). Movies were imported using an upsampling factor of 2, and frames were grouped into 31 fractions. Movie frames were aligned using patch motion correction with an output crop factor of 1/2 and B factor of 500 for global alignment. Micrograph contrast transfer function (CTF) estimation was performed using patch CTF estimation. Particle picking was first performed using a combination of Blob Picker followed by Template Picker with suitable classes selected after 2D classification of extracted particles. All particles were extracted using a box size of 512 pixels. All 2D classifications have an inner and outer mask diameter of 125 Å and 140 Å, respectively. A total of 186,280 particles were obtained after iterative 2D classification steps. An initial volume was generated through ab initio reconstruction of 158,100 particles with window inner and outer radius of 0.5 and 0.9, respectively, with octahedral symmetry imposed. Using this initial volume, Euler angles were refined for the 186,280 particles using homogeneous refinement with iterative refinements accounting for per-particle defocus optimization and per-group CTF parameters accounting for tilt, trefoil, spherical aberration, tetrafoil, and magnification anisotropy. A final refinement was performed accounting for Ewald sphere curvature correction using positive curvature. Refined particles, volume, and mask were then used for reference-based motion correction (RBMC) to optimize motion correction of particle images and for improved dose weighting (58,59). Finally, unbinned particles obtained from RBMC were re-refined to obtain a map at 1.33 Å global resolution. Random subsets of 180,000, 90,000, 45,000, 22,500, 11,250, 6000, 3000, 1500, 750, and 200 particles were selected using the Particle Subsets tool and subjected to homogeneous reconstructions to obtain maps resolved to progressively lower global resolutions as a function of decreasing particle number. Maps obtained from these reconstructions were used as inputs to derive confidence maps within the CCPEM software suite (60,61). A noise box of 192 pixels was used with an input pixel size of 0.228 and a box size of 1024. The false discovery rate Benjamini-Yekutieli (FDR-BY) correction method was employed using a two-sided test procedure. The rest of the parameters were default. Positions of waters were assessed at 0.1%, 1%, and 5% FDR. All visualizations were performed in Chimera or ChimeraX (University of California, San Francisco) (62,63).

RESULTS

In this section, we first compare water positions across nine high-resolution experimental apoferritin structures. We then present the development and evaluation of a method for predicting water positions around the apoferritin structure, based on calculating the excess chemical potential (WT).

High- and low-consensus water positions derived from the comparison of nine high-resolution experimental apoferritin structures

Prior to analyzing the statistical thermodynamic properties of interfacial water molecules, we compared the water positions across several experimental apoferritin structures. We obtained nine structures of apoferritin that were resolved by cryo-EM to 1.5 Å or higher (PDB: 8RQB (64), 7A6A (48), 8J5A (65), 7A4M (49), 6Z6U (48), 7RRP (12), 7A6B (48), 7K3V (12), and 7K3W (12)) (Table S1) from the Research Collaboratory for Structural Bioinformatics PDB (66). We then aligned these nine PDB structures to one another and identified a total of 1626 water molecules within 4 Å of a single apoferritin protomer when aggregated across all nine structures. Next, we grouped these positions into "clusters" by connecting any pair of water molecules less than 1 Å apart from one another and identified the disconnected sets of positions. For each cluster, the mean position of the water molecules was calculated. Clusters in which the mean position was more than 3.25 Å away from the apoferritin structure—the cutoff for the first hydration shell—were discarded, resulting in 196 unique water locations. The distribution of the number of clusters as a function of the number of water molecules per cluster is shown in Fig. 3. Water positions identified in five or more high-resolution cryo-EM structures were designated as high-consensus water positions, while those found in only 1–4 structures were classified as low-consensus water positions. Select high-consensus waters (red) and low-consensus waters (blue) are shown on the surface of the apoferritin monomer (Fig. 1 *B*). A higher proportion of low-consensus waters are located near the more flexible regions of apoferritin compared to the high-consensus waters.

Statistical thermodynamics of high- and low-consensus waters

To elucidate whether the differences between high- and low-consensus water positions can be attributed to the strength of the protein-water interactions, we calculated the total interaction energy of each of these water molecules with apoferritin. As shown in Fig. 4 *A*, the interaction energies of the high- and low-consensus water positions overlap, i.e., they are not well separated as a function of the interaction energy with the protein, which indicates that interaction energy with the protein alone does not sufficiently distinguish between these two groups. This underscores the need for alternative approaches to differentiate between high- and low-consensus water positions more effectively.

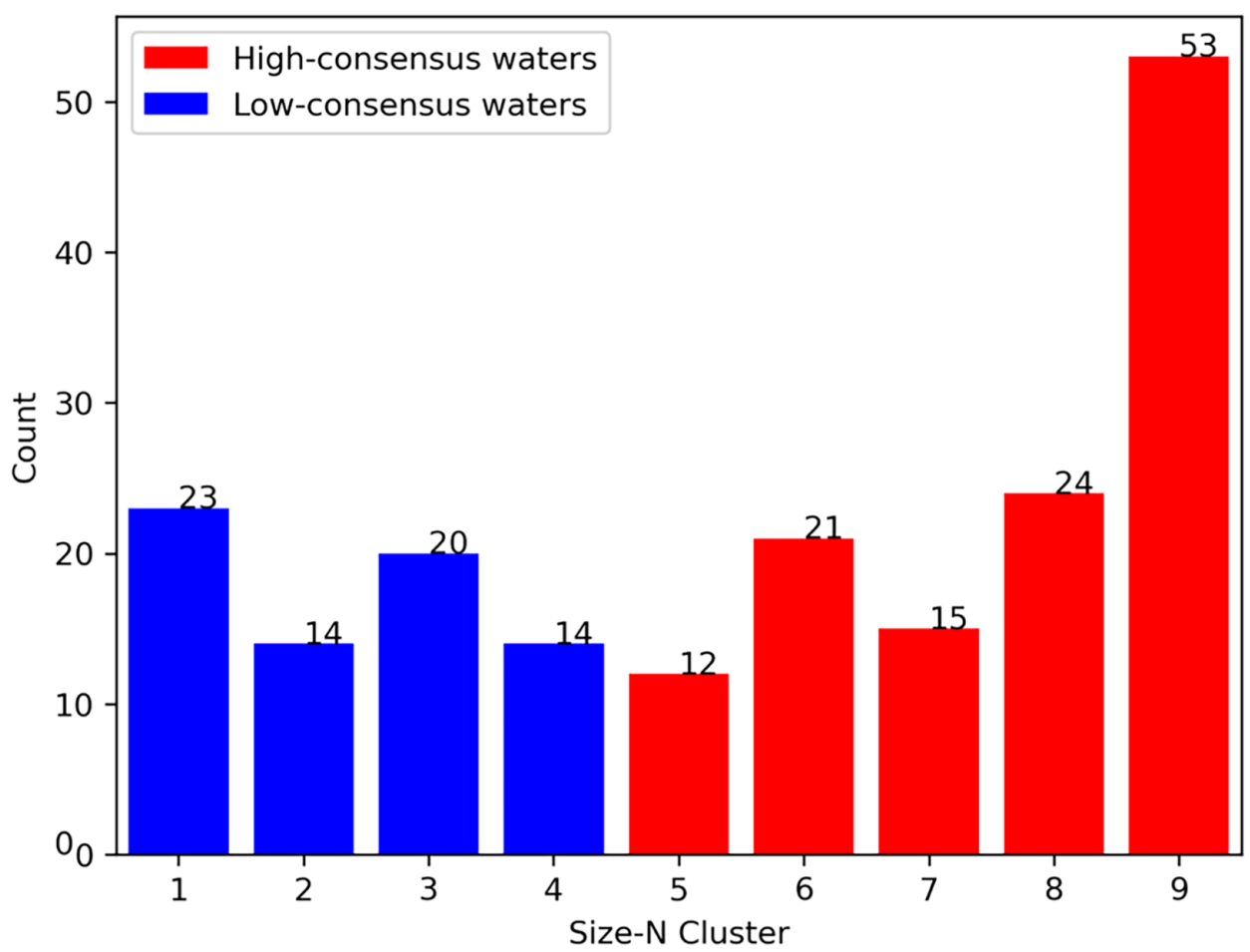

FIGURE 3 Water cluster sizes range from 1 to 9, with a size-9 cluster indicating that the water is present in all nine cryo-EM structures. The high-consensus water clusters (cluster size 5–9) are shown in red, and the low-consensus water clusters (cluster size 1–4) are shown in blue. These water molecules are within 3.25 Å of the apoferritin structure, ensuring proximity to the protein's surface. Additionally, they are separated by a minimum distance of 1 Å from one another to maintain spatial distinction and avoid overlapping density.

One such alternative method involves calculating the "excess chemical potential" (schematic diagram shown in Fig. 2), which quantifies the extent to which the presence of the protein alters the thermodynamic properties of water molecules compared to their behavior in bulk water. The excess chemical potential for a water molecule at position (**x**) is a statistical thermodynamic quantity that can be estimated by the difference between the solvation free energy for growing a water molecule at (**x**), $\Delta F(\mathbf{x})$, and the solvation free energy for growing a water molecule in the bulk, $\Delta F(\infty)$ (i.e., the pure liquid). The excess chemical potential is the "work to transfer" (WT) a water molecule from the bulk to the interfacial position at (**x**); it is proportional to the log ratio of the local density ($\rho(\mathbf{x})$) of water molecules at positions located at the interface of the protein to the bulk density ($\rho(\infty)$) (44). Details of the excess chemical potential (WT) calculations by the density ratio method are provided in materials and methods.

Using the MD simulation trajectory of a single apoferritin monomer solvated by ≈23,000 water molecules, we calculated the water density and the WT at the oxygen coordinates corresponding to each of the 196 unique water positions identified previously (see materials and methods). Our results, shown in Fig. 4, *A* and *B*, indicate that high-consensus water positions have significantly more favorable WT values compared to low-consensus water positions. This implies that waters at high-consensus positions are more localized and, therefore, the water density is higher than at low-consensus positions, according to Eq. 1. The WT values for high-consensus waters generally range from −3.5 to −4.8 kcal/mol, while for low-consensus waters WT values typically range from −1.2 to −3.5 kcal/mol. Of the 60 water locations with the most favorable WT values in Fig. 4 *B* (*gray shadow*), six correspond to a low-consensus water position, while 21 out of 60 water locations with the most favorable interaction energies (*gray shadow* in Fig. 4 *A*) correspond to low-consensus water positions. This distinction suggests that WT values are an effective metric for differentiating high-consensus waters from low-consensus waters on the surface of apoferritin.

Table S3 lists the excess chemical potential, the direct protein-water interaction energy, and the indirect free-energy component for each of the top 60 water locations ordered by excess chemical potential, WT (Fig. 4 *B*, *right*), and by average interaction energy, E_{p-w} (Fig. 4 *A*, *left*). The average chemical potential, direct interaction energy with

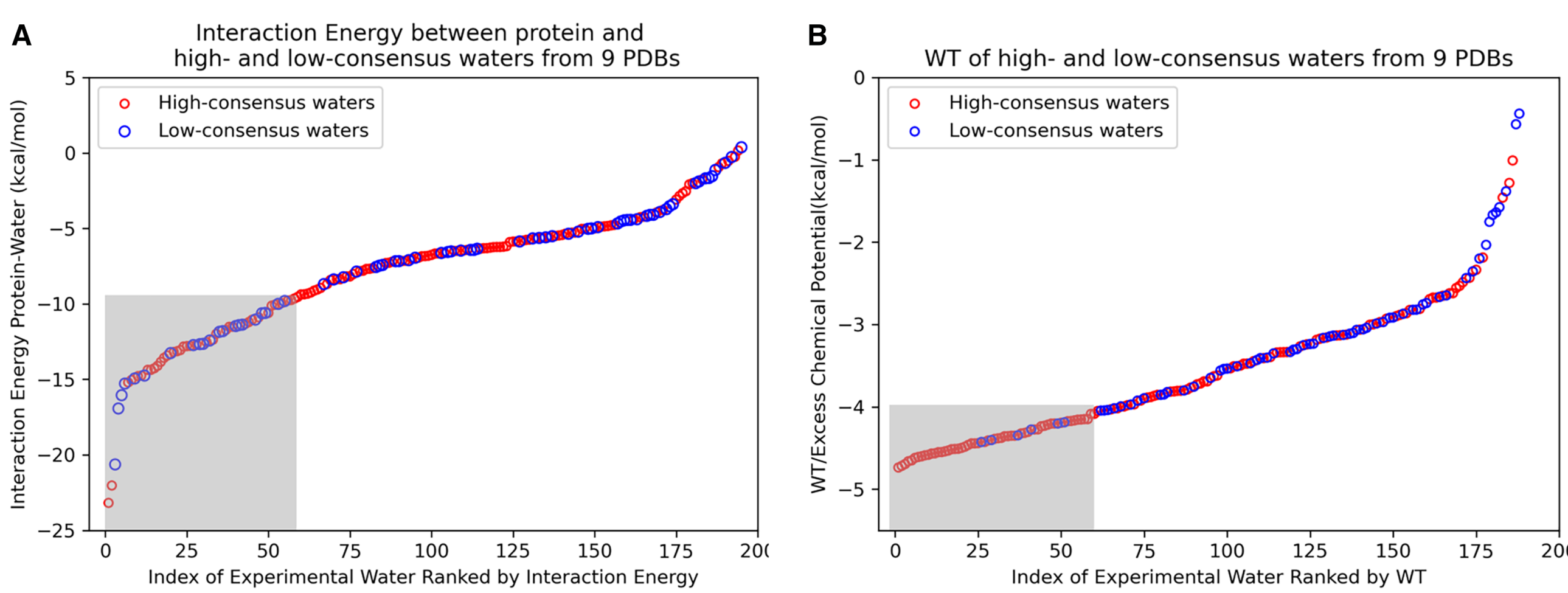

FIGURE 4 The interaction energy and WT between the protein and the 196 waters. (*A*) The interaction energy between the protein and high-consensus (*red*) and low-consensus (*blue*) water positions is shown. (*B*) Excess chemical potential (WT) values are illustrated for the high-consensus (*red*) and low-consensus (*blue*) water positions. The gray shaded areas represent the top 60 waters, ranked by interaction energy or WT values.

the protein, and indirect term for the 60 experimental water locations with the most favorable excess chemical potential WT = −4.4 ± 0.17 kcal/mol, E_{p-w} = −9.6 ± 4.0 kcal/mol, and ω = +5.2 ± 4.0 kcal/mol. The corresponding values for the top 60 experimental water locations ranked by the strength of the protein-water interaction energy are WT = −3.6 ± 1.0 kcal/mol, E_{p-w} = −12.8 ± 2.8 kcal/mol, and ω = +9.2 ± 3.0 kcal/mol. While the average excess chemical potentials for the two sets of water locations differs by less than 1 kcal/mol, the 60 water locations with the most favorable protein-water interaction energy are associated, on average, with a significantly more repulsive indirect contribution to the excess chemical potential than the 60 water locations with the most favorable excess chemical potentials (+9.2 kcal/mol vs. +5.2 kcal/mol). This indicates that the solvent structure in the environment close to the interfacial water locations with the strongest attraction to the protein surface are more perturbed relative to the bulk. We note also that there are many high-density interfacial water locations with very favorable excess chemical potential values that interact relatively weakly with the protein. For example, at the second most dense (WT = −4.70 kcal/mol) interfacial water location corresponding to a high-consensus position, the attractive interaction energy with the protein is only −5.4 kcal/mol; the opposing indirect term is also small (ω = +0.7 kcal/mol). In contrast, some of the low-consensus cryo-EM water locations are associated with locations with the strongest attractive interaction energies with the protein. The possible functional implications of these observations, as well as the implications for identifying in cryo-EM maps water locations that are less dense but interact very strongly with the protein, remain to be investigated.

Validation of predicted waters by comparison with experimental waters

We evaluated our method by assessing how well high- and low-consensus water positions are reproduced by calculating the distances between the WT-method-predicted water positions and those observed experimentally in apoferritin structures derived from cryo-EM. These experimental structures include nine apoferritin structures with resolutions higher than 1.5 Å (designated as group A PDBs) and 17 apoferritin structures with resolutions between 1.5 Å and 2.0 Å (designated as group B PDBs); details are provided in Table S1. The rationale for choosing 2.2 Å as the water match distance cutoff is severalfold: first, as shown in Fig. S1, the interaction between two hydrogen-bonded water molecules with optimal orientation is highly repulsive at 2.2 Å, indicating that predicted and experimental waters can be considered the same when they are within this distance. Second, this distance is the standard default recommendation for the lower distance limit to distinguish between individual solvent peaks within experimental protein refinement software, such as Phenix. Finally, previous studies have demonstrated that the cutoff distance for matching experimentally determined and predicted water positions ranges from 1.4 to 2.5 Å (18,67,68).

Fig. 5 shows the top 200 predicted water positions based on the most favorable excess chemical potential (WT) values. Of these, 92 and 29 waters are categorized as high consensus (*red solid circles*) and low consensus (*blue solid circles*), respectively, with an oxygen-oxygen match distance cutoff 2.2 Å. There are also 21 waters that are within 2.2 Å of the group B PDB water molecules (*red open diamonds*). The 58 remaining waters (*black stars*) are positioned outside the 2.2-Å cutoff of either group A or group B PDBs. The WT threshold was selected so that the number of predicted waters (200) closely matched the number of experimental waters (196). The 200 WT values range from −4.8 to −3.8 kcal/mol, corresponding to a density difference of less than 10-fold. However, the water density at these positions exceeds the bulk density by over 1000-fold. The large majority of the matched predicted water molecules are located within 1.0 Å of the experimental waters, as shown in Fig. S2. Among the 100 water locations with the most favorable excess chemical potentials, 85% are within 2.2 Å of a water molecule location identified in a cryo-EM map of 2.0 Å or higher resolution.

We calculated the precision and recall for predicted water positions across various excess chemical potential thresholds, as defined in materials and methods. Fig. 6, *A* and *B* illustrate the precision and recall for four datasets: high-consensus experimental water positions from group A PDBs (*red*), low-consensus experimental water positions from group A PDBs (*blue*), the sum of high-consensus and low-consensus positions from group A PDBs (*gray*), and the combined water positions from both group A and group B PDBs (*purple*).

In Fig. 6, the precision is notably higher for high-consensus waters (*red curve*) compared to low-consensus waters (*blue curve*). The accuracy of the predicted waters is particularly striking: approximately 70% of the top 200 predicted water positions (*purple square*), indexed by WT values, align with water positions observed in experimental apoferritin structures (match distance cutoff: 2.2 Å). This precision increases to 85% if we compare the 100 most densely populated waters predicted by the excess chemical potential to experimental waters (to the left of the *black vertical line* in Fig. 5). Additionally, as shown in Fig. 6, the excess chemical potential method successfully recovers 74% of the high-consensus water positions (*red square*). Using a 2.2-Å cutoff distance, 62% of the experimental waters resolved from group A PDBs (sum of high-consensus and low-consensus positions) are reproduced (*gray square*) when the WT threshold is set so that the number of predicted waters matches the experimental count (196).

Although most (>85%) of the predicted water positions with very favorable excess chemical potential values align

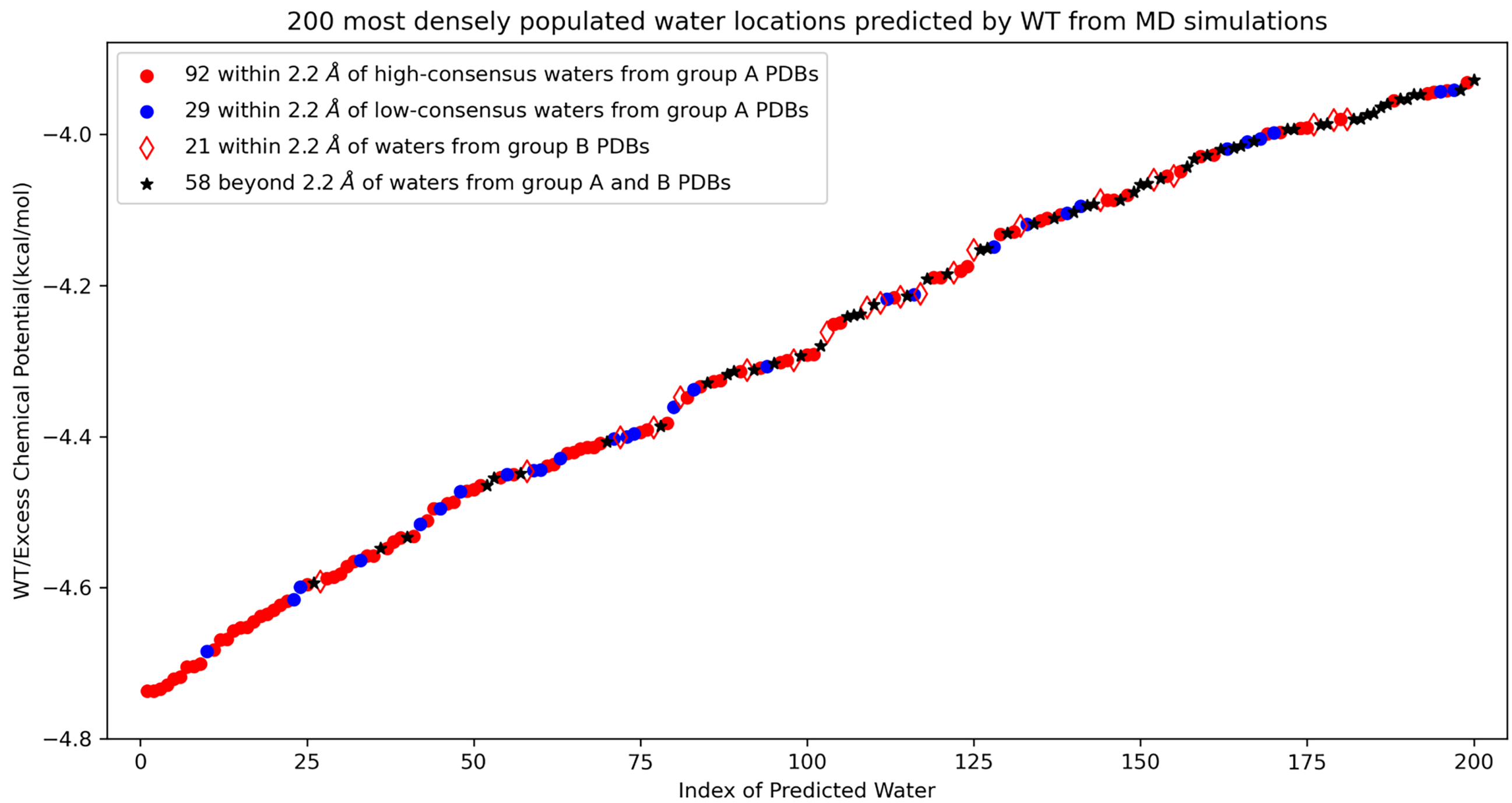

FIGURE 5 Among the 200 most densely populated water positions predicted by excess chemical potential (WT) from MD simulations, 92, 29, and 21 predicted waters are within a cutoff distance of 2.2 Å from high-consensus waters (*red solid circles*), low-consensus waters (*blue solid circles*), and group B PDBs (*red open diamonds*), respectively. The black star represents predicted waters that are not within 2.2 Å of any water molecules in group A or group B. Group A PDBs have resolutions higher than 1.5 Å, while group B PDBs have resolutions between 1.5 Å and 2.0 Å.

with those observed in cryo-EM structures, a small subset of these waters does not correspond to experimentally observed water molecules. For example, some waters appear to be caged by side chains of various apoferritin residues (Fig. 7). In the 100-ns MD trajectory, we observed that the caged waters exchange with surrounding waters at least 60 times, indicating that they are not trapped in the sites. This discrepancy may be attributed to several factors. 1) The pH, ion concentration, temperature, and other conditions during cryo-EM data collection differ from those used in the MD simulations. All these factors can contribute to protein hydration at specific locations, leading to discrepancies between experimental cryo-EM maps and MD simulations. 2) Because a single protomer is used in our MD simulations, some predicted water molecules overlap with the side chains of adjacent protomers, and waters at the interface of two or more protomers may not be accurately predicted. 3) Furthermore, in the MD simulations, the apoferritin protomer is fixed in position, which may impact the identification of waters near the flexible regions of the protein. 4) There is also the possibility that the water density in the cryo-EM reconstruction is weak, leading to its omission in model building and map annotation. The Chiu lab (35) has also identified additional reasons for the absence of MD-derived waters in experimental structures: 1) limitations of the classical force field in MD simulations, which might not fully capture polarization effects; and 2) differences in timescales between the methods—cryo-EM averages multiple chemical states over milliseconds during vitrification, while MD simulations capture a single state over much shorter, nanosecond to sub-millisecond, intervals (35).

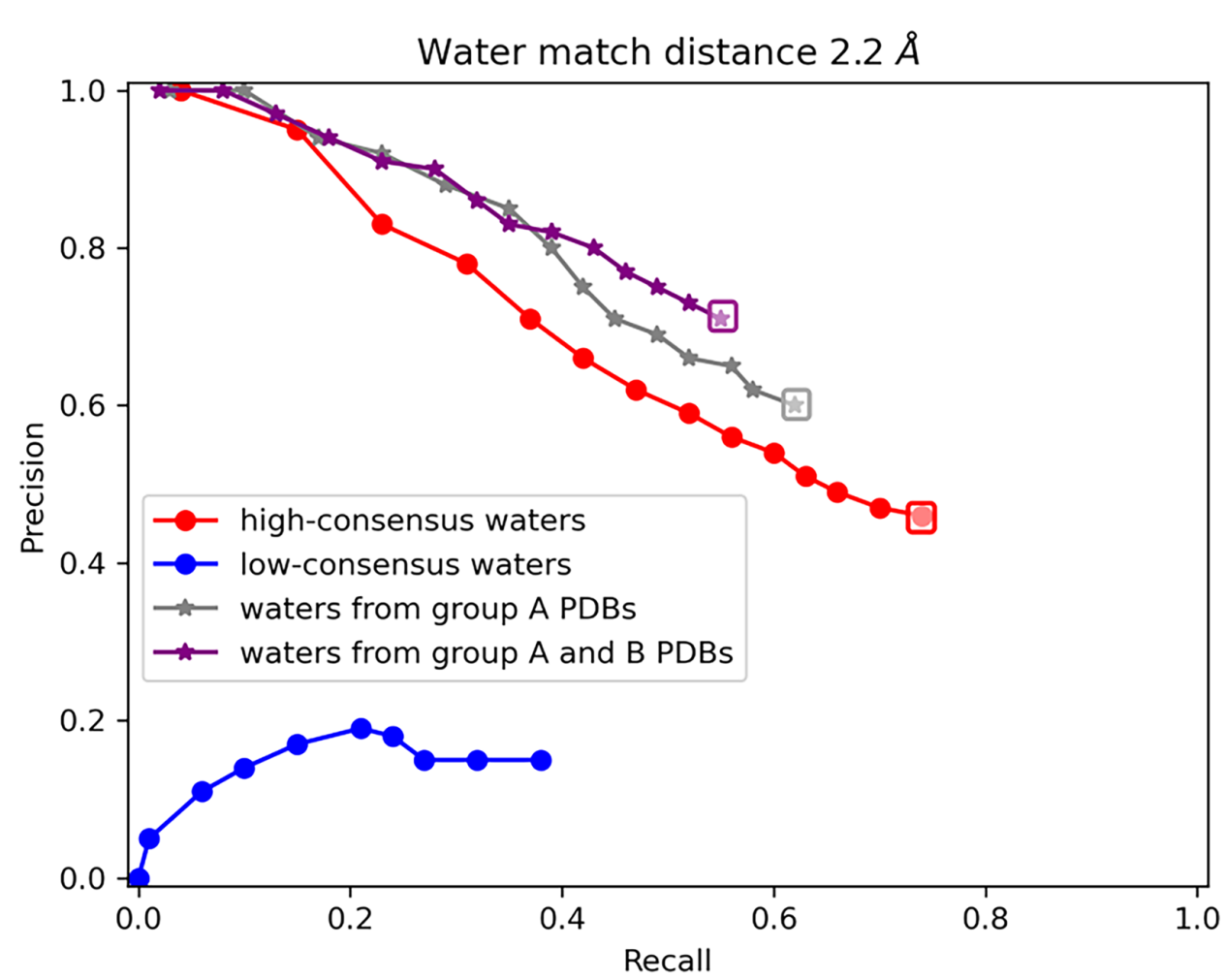

FIGURE 6 Precision-recall curves (PRC) are shown for the top 200 predicted water positions based on WT values with a 2.2-Å distance cutoff. The curves for high-consensus waters, low-consensus waters, their sum (experimental waters from group A PDBs), and the sum of all waters from group A and group B PDBs (all experimental waters) are presented in red, blue, gray, and purple, respectively.

Overall, our results demonstrate that the excess chemical potential of waters at the protein interface evaluated from

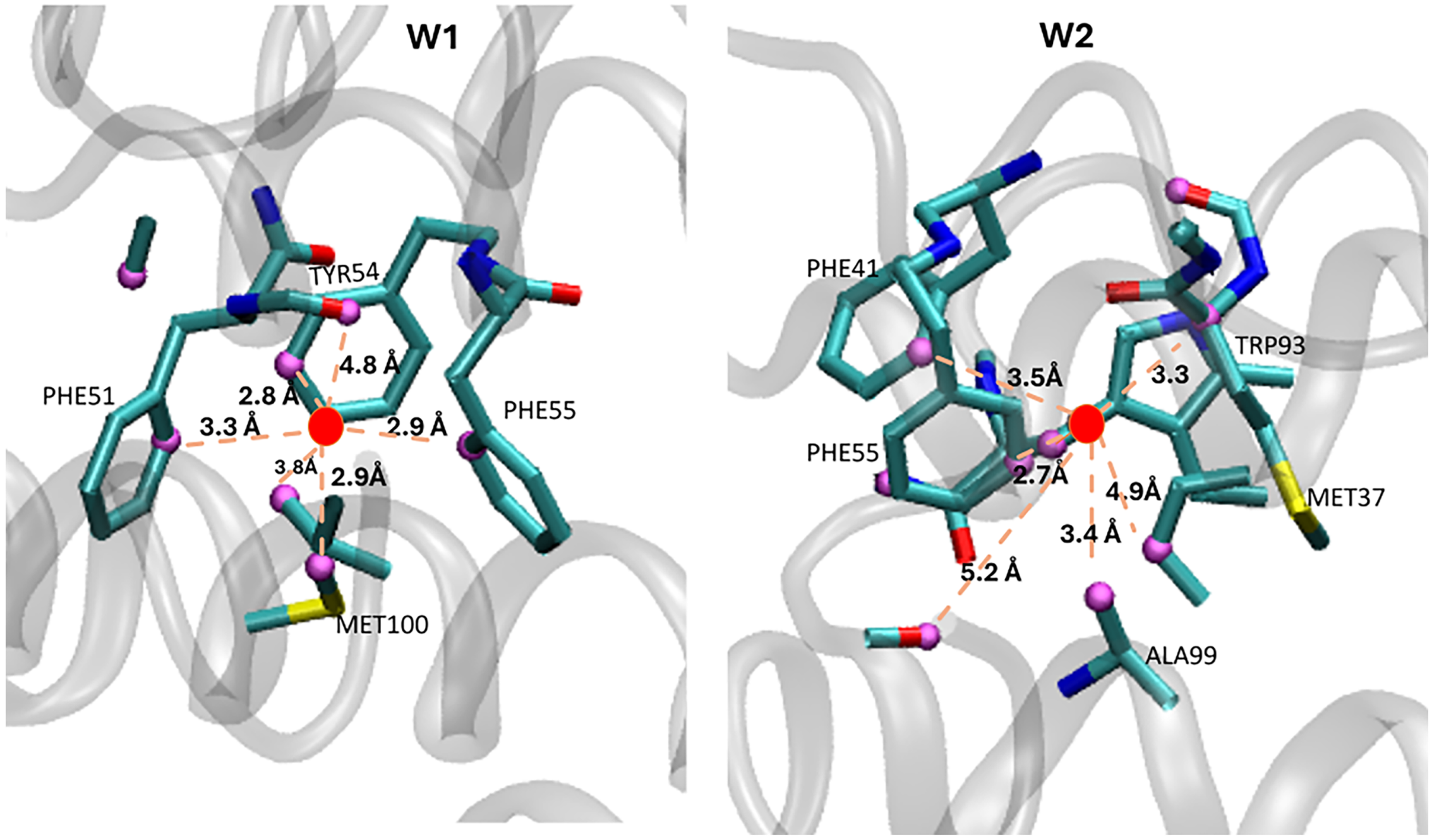

FIGURE 7 Two caged waters predicted from favorable excess chemical potential values are shown within the apoferritin structure. Red dots represent the oxygen atoms of caged water molecules, pink indicates residue atoms within 5 Å of the caged waters, and gray cartoon represents the apoferritin structure.

MD simulations can be effectively used to predict water positions in cryo-EM structures.

Analysis of the cryo-EM apoferritin structure using confidence maps at various resolutions

Following the identification of high-consensus and low-consensus water positions, we next assessed how the size, shape, and number of identified water molecules change as a function of resolution. To this end, we reprocessed a cryo-EM dataset of apoferritin (EMPIAR-10424, Electron Microscopy Data Bank 11638) and reconstructed maps using incrementally fewer particle numbers, yielding reconstructions at progressively lower resolution. We then calculated confidence maps derived from cryo-EM density maps at each of the resolutions. The confidence maps can be thresholded at different FDRs, which define the level of stringency applied when identifying water densities in the maps. Lower FDR values (1% or less) indicate higher confidence in the observed densities, whereas higher FDR values (above 1%) indicate lower confidence. Fig. S3 shows the cryo-EM apoferritin maps at various resolutions, each thresholded at the 1% FDR, as well as the density of Y168 side chain displayed at the bottom of each respective map. This figure helps to visualize how the map features vary as a function of particle numbers used in the final reconstruction. The detailed procedure for this analysis is provided in materials and methods.

All high-consensus water positions with strong WT values (−4.55 to −4.46 kcal/mol) that are consistently observed across all nine PDB structures display approximately spherical density distributions (Fig. 8, *A–C* at resolution 1.34 Å). By contrast, low-consensus water positions with weaker WT values (−3.76 to −3.31 kcal/mol) exhibit irregular and diffuse density distributions (Fig. 8, *D–F* at resolution 1.34 Å). High-consensus water positions remain clearly identifiable in confidence maps obtained from lower-resolution reconstructions (Fig. 8, *A–C* at resolution 2.21 Å), whereas low-consensus water positions exhibit greater variability across the resolution spectrum compared to high-consensus water positions (Fig. 8, *D–F* at resolution 1.67 Å; Table S2). Fig. S4 includes an expanded view that shows the surrounding residues, allowing the changes in water density across resolutions and thresholds to be visualized in the context of the local chemical environment.

Fig. 9 illustrates the number of observable waters per residue identified in the cryo-EM maps at the positions of the 196 high- and low-consensus waters (group A PDBs) that were thresholded at a 1% FDR as a function of resolution. The figure also includes a red line, which shows the predicted number of water molecules by WT. To determine this value, we used the experimental cryo-EM to benchmark and validate predicted positions from WT. To enable direct comparison with experimental data, the 200 waters with the highest WT values were selected, yielding a count closely matching the experimentally resolved group A waters (196). Among these, 142 positions overlap with

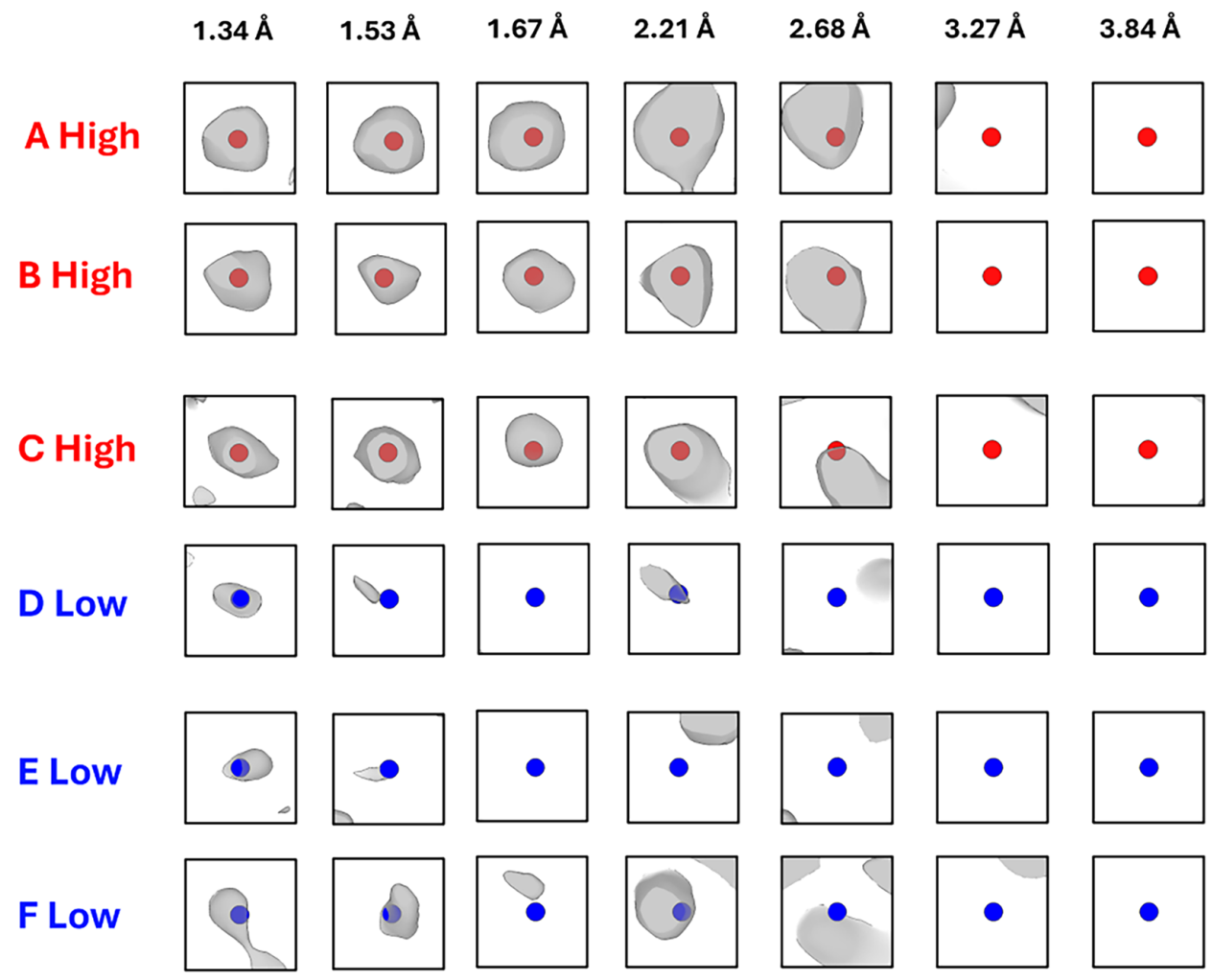

FIGURE 8 High-consensus (*A–C*) and low-consensus (*D–F*) waters are observed in confidence maps derived from cryo-EM data across a range of resolutions (1.34 Å, 1.53 Å, 1.67 Å, 2.21 Å, 2.68 Å, 3.27 Å, and 3.84 Å). These maps are displayed at a 1% false discovery rate threshold.

experimental waters (Fig. 5). Normalizing this number by the total apoferritin residue count produces the red curve (≈0.8) shown in Fig. 9. Notably, the predicted number of water molecules per apoferritin residue is independent of experimental resolution, as simulations were conducted using the PDB apoferritin structure without applying experimental density restraints to water. Our number of ≈0.8 waters per amino acid is consistent with previous estimates based on high-resolution data from the PDB. For example, Biedermannová and Schneider suggested a water/amino acid ratio of ≈0.9 (69). Nittinger et al. indicated a large variability in the water/amino acid content that is dependent on the protein but also found that on average this number is ≈0.75:1 (with a median value of ≈1:1) (70).

The inclusion of water molecules in cryo-EM models is highly dependent on the resolution of the structure. Extrapolating the curve in Fig. 9, it is apparent that the resolution of the experimental structure needs to be ≈1.0 Å to resolve all the 0.8 water molecules per residue in apoferritin. Again, the number of waters in the cryo-EM apoferritin model (PDB: 7A4M) based on this extrapolation is consistent with estimates from deposited high-resolution X-ray crystal structures, which indicate ≈0.9 ± 0.5 water molecules per protein residue (69). As the resolution of the experimental maps decreases, so does the number of confidently assigned water molecules. At the 1.34-Å resolution corresponding to our best experimental reconstruction, we observe ≈0.65 water molecules per residue. At resolutions of 2.0 Å, 2.5 Å, and 3.0 Å, we observe ≈0.4, ≈0.2, and ≈0.1 water molecules per residue, respectively. Below a resolution of ≈3.25 Å, water molecules are no longer identifiable. This trend is similar to that of a prior study of apoferritin cryo-EM structures, where density associated with a water molecule is observed at 2.3 Å but not at 3.1 Å (71). The results from visualizing maps at 0.1% and 5% FDR thresholds are shown in Fig. S5. Despite varying levels of stringency between confidence maps visualized at 0.1%, 1%, and 5% FDR, the trends of observing progressively fewer waters with decreasing resolution are all similar. This result demonstrates quantitatively how resolution limitations can lead to ambiguities and challenges when determining positions of waters based on cryo-EM maps.

Fig. 10 illustrates the reliability scores that we have derived based on confidence maps calculated from cryo-EM maps at two different resolutions: 2.7 Å and 1.3 Å. Since cryo-EM maps do not have a uniform scale for thresholding, we have used confidence maps to ensure the thresholding is uniform between maps of different resolution. Water positions at high resolution (1.3 Å) are much more likely to have a reliability score of 1, indicating that the water is clearly observed at that location in the cryo-EM map. By contrast, a reliability score of 0 indicates that the water is not observed at an FDR of 1% (see discussion), while a reliability score of 0.5 reflects ambiguous or uncertain density for that water. Similarly, Table 1 shows a strong tendency for waters to be assigned a reliability score of 1 at 1.3-Å resolution (109 out of 128 instances). At 2.7-Å resolution, however, waters are more frequently assigned a reliability score of 0 or 0.5 (104 instances) compared to a score of 1 (24 instances). These findings confirm that the number of experimentally observed water molecules strongly depends on the resolution of the cryo-EM map. We believe the excess chemical potential method described in this study can thus be a powerful resolution-agnostic tool to inform about potential water positions. In future work, we plan to explore approaches that integrate reliability scores derived from cryo-EM maps with excess chemical potential values obtained from MD simulations to identify highly probable water positions—particularly those with intermediate reliability scores (e.g., 0.5)—in lower-resolution cryo-EM maps.

DISCUSSION

Accurate water assignment in cryo-EM maps remains error prone, especially in cryo-EM maps that are resolved to resolutions poorer than 2 Å. Given the challenges faced by experimentalists, it is important to develop diverse tools to assign—and validate—water positions. Although deep-learning approaches can help automate water assignments, distinguish waters from metal ions or other species, and provide confidence measures based on prior data, the fundamental limitation of all current approaches is reliance on accurate training data. The often "black-box" nature of machine-learning approaches, and their reliance on large and potentially limiting training datasets, implies that physical insight into new positions may be missed. For instance, a recent report highlighted how treating experimental cryo-EM maps with sharpening tools based on deep neural

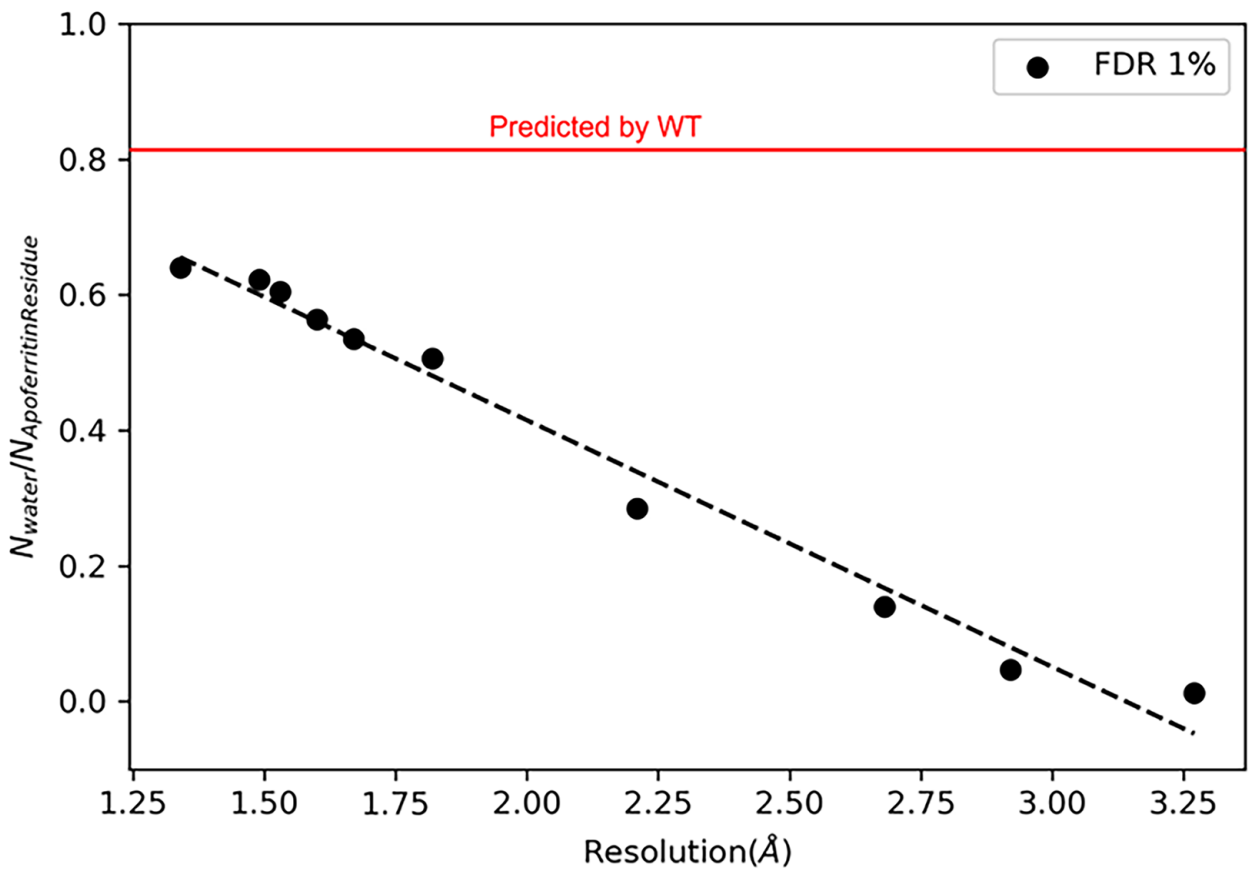

FIGURE 9 The number of water molecules per apoferritin residue is plotted as a function of map resolution. Data analyzed on confidence maps at 1% false discovery rate (FDR) threshold.

networks can attenuate, or completely eliminate, experimental densities corresponding to ligands and solvent (72). In this regard, the major advantage of our current method is the ability to improve water identification within cryo-EM density maps in a resolution-agnostic manner, based on physical principles that are rooted in statistical thermodynamics.

Our study demonstrates the effectiveness of using statistical thermodynamic properties of water to identify interfacial waters in cryo-EM structures of apoferritin. Moreover, our findings revealed that the average interaction energy with the protein is insufficient for effectively differentiating between high- and low-consensus water positions. By utilizing WT values derived from MD simulations, we can better distinguish between high- and low-consensus water locations. We note the strong prediction performance of our method based on the evaluation of the excess chemical potential, which requires integration of the local water density over a finite volume using an ultra-fine-grained grid for the integration. At a recall rate of 55%, we achieved a precision of 71% for waters from group A + B PDBs. At the same recall rate, our method outperforms HydraProt (18) (precision 55%). Our approach also exhibits higher precision when compared to a similar MD-based approach that attempted to identify individual positions of peak water density in a fine grained grid (35), where only ≈25% of waters were identified as true positives. Also, we found a strong correlation between WT values and reliability scores derived from the confidence map of a 1.3-Å reconstruction. Some waters with very favorable WT values are not observed in the experimental structures, particularly waters that appear to be “caged” by surrounding aromatic side chains. Further analysis and experimentation with variation of density thresholding may be helpful in resolving these discrepancies.

The number of observable waters in cryo-EM models strongly depends on the resolution of the structure. At resolutions below 3 Å, it becomes increasingly challenging to identify water densities, although the most tightly bound waters appear to be identifiable even down to resolutions of ≈3.25 Å. At these lower resolutions, water features may either blend with noise or become entirely indistinguishable. Most cryo-EM structures are resolved to between ≈2 Å and 4 Å (73). For the lower-resolution structures, water densities may thus be difficult or impossible to resolve. As shown in Fig. 9, waters that are clearly visible at 1.3 Å largely disappear or become too noisy to distinguish at 2.7 Å. In such scenarios, our WT method can provide a significant advantage by accurately predicting water positions and distinguishing water densities from noise. This approach not only aids modeling but also accelerates the placement of waters in low-resolution cryo-EM structures, offering valuable insights into structural interpretation. Furthermore, we note that our MD simulations are performed without experimental density restraints, yet the water positions with very favorable WT values correlate

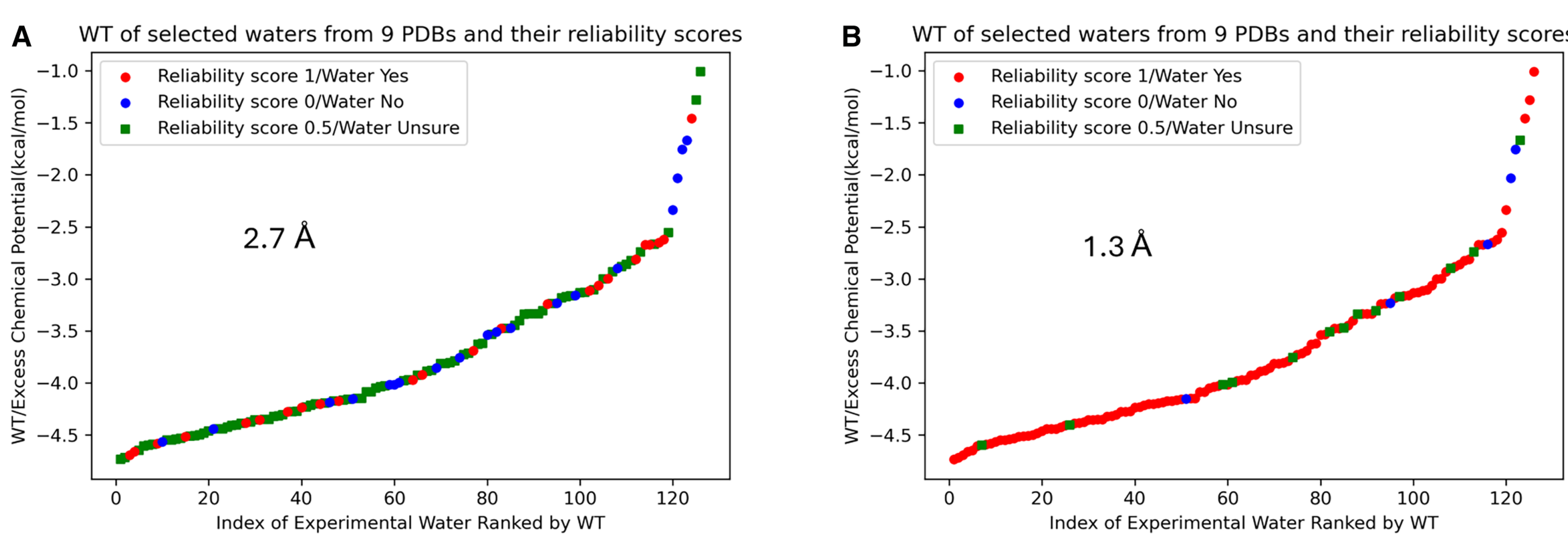

FIGURE 10 The excess chemical potential (WT) values are plotted for two different resolutions: (*A*) 2.7 Å and (*B*) 1.3 Å. Water molecules are colored based on their reliability scores: red for waters with a score of 1 (clearly resolved density), blue for a score of 0 (no observable density), and green for a score of 0.5 (ambiguous or uncertain density). Data analyzed on confidence maps at 1% FDR threshold.

TABLE 1 Number of waters identified at two different resolutions (2.7 Å and 1.3 Å) as a function of reliability score

	Reliability score			Total
	1	0	0.5	
Density	yes	no	noisy	–
2.7 Å	24	19	85	128
1.3 Å	109	5	14	128

strongly with their corresponding position within experimentally defined maps. This indicates that such tools would likely be useful to boost confidence in annotated water positions and to identify new water positions and correlated networks of waters that are associated with allostery, based on physical principles and thermodynamic signatures. These ideas can also be extended to identifying and characterizing metal ions within experimental maps. As a specific case study, in maps of lentiviral intasomes, the same peak within the active site of the ligand-bound complex was identified as a chloride ion in one structure (5) but a water molecule in another analogous structure (1). By using the excess chemical potential (WT) of water calculated from MD simulations and estimating the excess chemical potential of the ion, it may be possible to distinguish between water and ions at specific locations by comparing their WT values evaluated by a procedure involving post-processing of the trajectories to assign the relative likelihood of water versus ions being located at specific sites.

Approaches that we are developing will build upon the current framework but will also include considerations of local resolution, density restraints to guide the MD simulations, integration of information concerning water locations from cryo-EM confidence maps with maps of the solvent excess chemical potential generated from MD simulations, and iterative model building and density-guided refinement approaches for optimal hydration of experimental cryo-EM maps.

DATA AND CODE AVAILABILITY

Data are contained within the article and supporting material. Additional data will be shared upon reasonable request.

ACKNOWLEDGMENTS

One of the authors (R.M.L.) is especially pleased to contribute this paper to the special issue of *Biophysical Journal* honoring the many contributions of Martin Karplus to biophysics & structural biology and theoretical & computational chemistry. Martin always emphasized the importance of establishing close connections between theory, computation, and experimentation. R.M.L. worked with Martin in the late 1970s and early 1980s to establish connections between some of the first protein MD simulations and corresponding NMR and X-ray experiments that could probe very fast protein motions on picosecond timescales. This contribution reflects some of the lessons that R.M.L. learned from Martin Karplus and is in the spirit of their work together more than 40 years ago. This study is primarily supported by the National Institutes of Health (NIH) grant R01 AI178849 (to R.M.L. and D.L.). D.L. is also supported by NIH grants U01 AI136680 (to D.L. and R.M.L.), R01 AI146017, and U54 AI170855 (to D.L. and R.M.L.) as well as the Margaret T. Morris Foundation and the Hearst Foundations. A.B. was supported by the Eric & Wendy Schmidt AI-in-Science Postdoctoral Fellowship, a program of Schmidt Sciences. N.M. is grateful to Grants-in-Aid for Scientific Research (nos. JP23H02622 and JP23K27313) from the Japan Society for the Promotion of Science; the Fugaku Supercomputer Project (nos. JPMXP1020230325 and JPMXP1020230327) and the Data-Driven Material Research Project (no. JPMXP1122714694) from the Ministry of Education, Culture, Sports, Science and Technology; and the Maruho Collaborative Project for Theoretical Pharmaceutics. Explicit solvent MD simulations were run on the local computing resource, the CB2RR HPC cluster at Temple University, and the Expanse clusters of ACCESS (MCB100145). The WT calculations were run on the CB2RR HPC cluster using custom Python scripts.

AUTHOR CONTRIBUTIONS

Designed research, R.M.L., N.M., and D.L.; performed research, Q.S., S.A., A.B., and S.C.; contributed analytic tools, Q.S., A.H., S.A., and A.B.; resources, R.M.L. and D.L.; data analysis, Q.S., S.A., A.B., and S.C.; writing – original draft preparation, Q.S. and S.C.; writing – review & editing, D.L., S.A., A.B., and R.M.L.; supervision, D.L. and R.L.; funding acquisition, R.M.L., N.M., A.B., and D.L. All authors have read and agreed to the published version of the manuscript.

DECLARATION OF INTERESTS

The authors declare no competing interests.

SUPPORTING MATERIAL

Supporting material can be found online at https://doi.org/10.1016/j.bpj.2026.01.043.

REFERENCES

1. Passos, D. O., M. Li, …, D. Lyumkis. 2020. Structural basis for strand-transfer inhibitor binding to HIV intasomes. *Science.* 367:810–814. https://doi.org/10.1126/science.aay8015. https://www.ncbi.nlm.nih.gov/pubmed/32001521.
2. Schiebel, J., R. Gaspari, …, G. Klebe. 2018. Intriguing role of water in protein-ligand binding studied by neutron crystallography on trypsin complexes. *Nat. Commun.* 9:3559. https://doi.org/10.1038/s41467-018-05769-2. https://www.ncbi.nlm.nih.gov/pubmed/30177695.
3. Biela, A., F. Sielaff, …, G. Klebe. 2012. Ligand binding stepwise disrupts water network in thrombin: enthalpic and entropic changes reveal classical hydrophobic effect. *J. Med. Chem.* 55:6094–6110. https://doi.org/10.1021/jm300337q. https://www.ncbi.nlm.nih.gov/pubmed/22612268.
4. Cui, D., B. W. Zhang, …, R. M. Levy. 2018. The Role of Interfacial Water in Protein-Ligand Binding: Insights from the Indirect Solvent Mediated Potential of Mean Force. *J. Chem. Theory Comput.* 14:512–526. https://doi.org/10.1021/acs.jctc.7b01076.
5. Cook, N. J., W. Li, …, P. Cherepanov. 2020. Structural basis of second-generation HIV integrase inhibitor action and viral resistance. *Science.* 367:806–810. https://doi.org/10.1126/science.aay4919. https://www.ncbi.nlm.nih.gov/pubmed/32001525.
6. Li, M., D. Oliveira Passos, …, D. Lyumkis. 2023. Mechanisms of HIV-1 integrase resistance to dolutegravir and potent inhibition of drug-resistant variants. *Sci. Adv.* 9:eadg5953. https://doi.org/10.1126/sciadv.adg5953. https://www.ncbi.nlm.nih.gov/pubmed/37478179.
7. Poornima, C. S., and P. M. Dean. 1995. Hydration in drug design. 1. Multiple hydrogen-bonding features of water molecules in mediating

protein-ligand interactions. *J. Comput. Aided Mol. Des.* 9:500–512. https://doi.org/10.1007/bf00124321.

8. Wong, S. E., and F. C. Lightstone. 2011. Accounting for water molecules in drug design. *Expert Opin. Drug Discov.* 6:65–74. https://doi.org/10.1517/17460441.2011.534452.

9. Laage, D., T. Elsaesser, and J. T. Hynes. 2017. Water Dynamics in the Hydration Shells of Biomolecules. *Chem. Rev.* 117:10694–10725. https://doi.org/10.1021/acs.chemrev.6b00765.

10. Levy, Y., and J. N. Onuchic. 2006. Water mediation in protein folding and molecular recognition. *Annu. Rev. Biophys. Biomol. Struct.* 35:389–415. https://doi.org/10.1146/annurev.biophys.35.040405.102134.

11. Mitusinska, K., A. Raczynska, …, A. Gora. 2020. Applications of water molecules for analysis of macromolecule properties. *Comput. Struct. Biotechnol. J.* 18:355–365. https://doi.org/10.1016/j.csbj.2020.02.001. https://www.ncbi.nlm.nih.gov/pubmed/32123557.

12. Zhang, K., G. D. Pintilie, …, W. Chiu. 2020. Resolving individual atoms of protein complex by cryo-electron microscopy. *Cell Res.* 30:1136–1139. https://doi.org/10.1038/s41422-020-00432-2.

13. Liebschner, D., P. V. Afonine, …, P. D. Adams. 2019. Macromolecular structure determination using X-rays, neutrons and electrons: recent developments in Phenix. *Acta Crystallogr. D Struct. Biol.* 75:861–877. https://doi.org/10.1107/S2059798319011471.

14. Yamashita, K., C. M. Palmer, …, G. N. Murshudov. 2021. Cryo-EM single-particle structure refinement and map calculation using Servalcat. *Acta Crystallogr. D Struct. Biol.* 77:1282–1291. https://doi.org/10.1107/S2059798321009475. https://www.ncbi.nlm.nih.gov/pubmed/34605431.

15. Wang, J., Z. Liu, …, P. B. Moore. 2018. Identification of ions in experimental electrostatic potential maps. *IUCrJ.* 5:375–381. https://doi.org/10.1107/S2052252518006292. https://www.ncbi.nlm.nih.gov/pubmed/30002838.

16. Marques, M. A., M. D. Purdy, and M. Yeager. 2019. CryoEM maps are full of potential. *Curr. Opin. Struct. Biol.* 58:214–223. https://doi.org/10.1016/j.sbi.2019.04.006. https://www.ncbi.nlm.nih.gov/pubmed/31400843.

17. Prisant, M. G., C. J. Williams, …, D. C. Richardson. 2020. New tools in MolProbity validation: CaBLAM for CryoEM backbone, UnDowser to rethink "waters," and NGL Viewer to recapture online 3D graphics. *Protein Sci.* 29:315–329. https://doi.org/10.1002/pro.3786. https://www.ncbi.nlm.nih.gov/pubmed/31724275.

18. Zamanos, A., G. Ioannakis, and I. Z. Emiris. 2024. HydraProt: A New Deep Learning Tool for Fast and Accurate Prediction of Water Molecule Positions for Protein Structures. *J. Chem. Inf. Model.* 64:2594–2611. https://doi.org/10.1021/acs.jcim.3c01559.

19. Park, S., and C. Seok. 2022. GalaxyWater-CNN: Prediction of Water Positions on the Protein Structure by a 3D-Convolutional Neural Network. *J. Chem. Inf. Model.* 62:3157–3168. https://doi.org/10.1021/acs.jcim.2c00306. https://www.ncbi.nlm.nih.gov/pubmed/35749367.

20. Kawama, K., Y. Fukushima, …, T. Yoshidome. 2022. gr Predictor: A Deep Learning Model for Predicting the Hydration Structures around Proteins. *J. Chem. Inf. Model.* 62:4460–4473. https://doi.org/10.1021/acs.jcim.2c00987. https://www.ncbi.nlm.nih.gov/pubmed/36068974.

21. Shub, L., W. Liu, …, M. J. Robertson. 2025. MIC: A deep learning tool for assigning ions and waters in cryo-EM and crystal structures. *Nat. Commun.* 16:6182. https://doi.org/10.1038/s41467-025-61315-x. https://www.ncbi.nlm.nih.gov/pubmed/40615379.

22. Westhof, E., B. Chevrier, …, R. M. Levy. 1986. Temperature-dependent molecular dynamics and restrained X-ray refinement simulations of a Z-DNA hexamer. *J. Mol. Biol.* 191:699–712. https://doi.org/10.1016/0022-2836(86)90454-7. https://www.ncbi.nlm.nih.gov/pubmed/3806679.

23. Kuriyan, J., G. A. Petsko, …, M. Karplus. 1986. Effect of anisotropy and anharmonicity on protein crystallographic refinement. An evaluation by molecular dynamics. *J. Mol. Biol.* 190:227–254. https://doi.org/10.1016/0022-2836(86)90295-0. https://www.ncbi.nlm.nih.gov/pubmed/3795269.

24. Levy, R. M., C. M. Dobson, and M. Karplus. 1982. Dipolar NMR relaxation of nonprotonated aromatic carbons in proteins. Structural and dynamical effects. *Biophys. J.* 39:107–113. https://doi.org/10.1016/S0006-3495(82)84496-2. https://www.ncbi.nlm.nih.gov/pubmed/6179550.

25. Makarov, V. A., B. K. Andrews, …, B. M. Pettitt. 2000. Residence times of water molecules in the hydration sites of myoglobin. *Biophys. J.* 79:2966–2974. https://doi.org/10.1016/S0006-3495(00)76533-7. https://www.ncbi.nlm.nih.gov/pubmed/11106604.

26. Phillips, G. N., Jr., and B. M. Pettitt. 1995. Structure and dynamics of the water around myoglobin. *Protein Sci.* 4:149–158. https://doi.org/10.1002/pro.5560040202. https://www.ncbi.nlm.nih.gov/pubmed/7757005.

27. Gelpi, J. L., S. G. Kalko, …, M. Orozco. 2001. Classical molecular interaction potentials: improved setup procedure in molecular dynamics simulations of proteins. *Proteins.* 45:428–437. https://doi.org/10.1002/prot.1159. https://www.ncbi.nlm.nih.gov/pubmed/11746690.

28. Malham, R., S. Johnstone, …, S. W. Homans. 2005. Strong solute-solute dispersive interactions in a protein-ligand complex. *J. Am. Chem. Soc.* 127:17061–17067. https://doi.org/10.1021/ja055454g. https://www.ncbi.nlm.nih.gov/pubmed/16316253.

29. Papoian, G. A., J. Ulander, …, P. G. Wolynes. 2004. Water in protein structure prediction. *Proc. Natl. Acad. Sci. USA.* 101:3352–3357. https://doi.org/10.1073/pnas.0307851100. https://www.ncbi.nlm.nih.gov/pubmed/14988499.

30. Young, T., R. Abel, …, R. A. Friesner. 2007. Motifs for molecular recognition exploiting hydrophobic enclosure in protein-ligand binding. *Proc. Natl. Acad. Sci. USA.* 104:808–813. https://doi.org/10.1073/pnas.0610202104. https://www.ncbi.nlm.nih.gov/pubmed/17204562.

31. Resat, H., and M. Mezei. 1994. Grand Canonical Monte Carlo Simulation of Water Positions in Crystal Hydrates. *J. Am. Chem. Soc.* 116:7451–7452. https://doi.org/10.1021/ja00095a076.

32. Speidel, J. A., J. R. Banfelder, and M. Mezei. 2006. Automatic Control of Solvent Density in Grand Canonical Ensemble Monte Carlo Simulations. *J. Chem. Theory Comput.* 2:1429–1434. https://doi.org/10.1021/ct0600363. https://www.ncbi.nlm.nih.gov/pubmed/26626850.

33. Hamelberg, D., and J. A. McCammon. 2004. Standard free energy of releasing a localized water molecule from the binding pockets of proteins: double-decoupling method. *J. Am. Chem. Soc.* 126:7683–7689. https://doi.org/10.1021/ja0377908. https://www.ncbi.nlm.nih.gov/pubmed/15198616.

34. Barillari, C., J. Taylor, …, J. W. Essex. 2007. Classification of water molecules in protein binding sites. *J. Am. Chem. Soc.* 129:2577–2587. https://doi.org/10.1021/ja066980q. https://www.ncbi.nlm.nih.gov/pubmed/17288418.

35. Roh, S.-H., M. Shekhar, …, W. Chiu. 2020. Cryo-EM and MD infer water-mediated proton transport and autoinhibition mechanisms of V(o) complex. *Sci. Adv.* 6:eabb9605. https://doi.org/10.1126/sciadv.abb9605.

36. Kretsch, R. C., S. Li, …, W. Chiu. 2025. Complex water networks visualized by cryogenic electron microscopy of RNA. *Nature.* 642:250–259. https://doi.org/10.1038/s41586-025-08855-w. https://www.ncbi.nlm.nih.gov/pubmed/40068818.

37. Chandler, D. 2005. Interfaces and the driving force of hydrophobic assembly. *Nature.* 437:640–647. https://doi.org/10.1038/nature04162.

38. Tanford, C. 1997. How protein chemists learned about the hydrophobic factor. *Protein Sci.* 6:1358–1366. https://doi.org/10.1002/pro.5560060627.

39. Rasaiah, J. C., S. Garde, and G. Hummer. 2008. Water in Nonpolar Confinement: From Nanotubes to Proteins and Beyond. *Annu. Rev. Phys. Chem.* 59:713–740. https://doi.org/10.1146/annurev.physchem.59.032607.093815.

40. Striolo, A. 2016. Interfacial water studies and their relevance for the energy sector. *Mol. Phys.* 114:2615–2626. https://doi.org/10.1080/00268976.2016.1237685.

41. Michel, J., J. Tirado-Rives, and W. L. Jorgensen. 2009. Prediction of the Water Content in Protein Binding Sites. *J. Phys. Chem. B.* 113:13337–13346. https://doi.org/10.1021/jp9047456.

42. Wang, L., R. A. Friesner, and B. J. Berne. 2011. Correction to "Replica Exchange with Solute Scaling: A More Efficient Version of Replica Exchange with Solute Tempering (REST2)". *J. Phys. Chem. B.* 115:11305.

43. Nguyen, C. N., T. K. Young, and M. K. Gilson. 2012. Grid inhomogeneous solvation theory: Hydration structure and thermodynamics of the miniature receptor cucurbit[7]uril. *J. Chem. Phys.* 137:044101. https://doi.org/10.1063/1.4733951.

44. Zhang, B. W., D. Cui, …, R. M. Levy. 2018. The Excess Chemical Potential of Water at the Interface with a Protein from End Point Simulations. *J. Phys. Chem. B.* 122:4700–4707. https://doi.org/10.1021/acs.jpcb.8b02666.

45. Widom, B. 1963. Some topics in the theory of fluids. *J. Chem. Phys.* 39:2808–2812.

46. Widom, B. 1982. Potential-distribution theory and the statistical mechanics of fluids. *J. Phys. Chem.* 86:869–872.

47. Hamdi, F., C. Tüting, …, P. L. Kastritis. 2020. 2.7 Å cryo-EM structure of vitrified M. musculus H-chain apoferritin from a compact 200 keV cryo-microscope. *PLoS One.* 15:e0232540. https://doi.org/10.1371/journal.pone.0232540.

48. Yip, K. M., N. Fischer, …, H. Stark. 2020. Atomic-resolution protein structure determination by cryo-EM. *Nature.* 587:157–161. https://doi.org/10.1038/s41586-020-2833-4. https://www.ncbi.nlm.nih.gov/pubmed/33087927.

49. Nakane, T., A. Kotecha, …, S. H. W. Scheres. 2020. Single-particle cryo-EM at atomic resolution. *Nature.* 587:152–156. https://doi.org/10.1038/s41586-020-2829-0. https://www.ncbi.nlm.nih.gov/pubmed/33087931.

50. Parrinello, M., and A. Rahman. 1981. Polymorphic transitions in single crystals: A new molecular dynamics method. *J. Appl. Phys.* 52:7182–7190.

51. Essmann, U., L. Perera, …, L. G. Pedersen. 1995. A Smooth Particle Mesh Ewald Method. *J. Chem. Phys.* 103:8577–8593.

52. Levy, R. M., D. Cui, …, N. Matubayasi. 2017. Relationship between Solvation Thermodynamics from IST and DFT Perspectives. *J. Phys. Chem. B.* 121:3825–3841. https://doi.org/10.1021/acs.jpcb.6b12889.

53. Matubayasi, N., and M. Nakahara. 2000. Theory of solutions in the energetic representation. I. Formulation. *J. Chem. Phys.* 113:6070–6081. https://doi.org/10.1063/1.1309013.

54. Matubayasi, N., and M. Nakahara. 2002. Theory of solutions in the energy representation. II. Functional for the chemical potential. *J. Chem. Phys.* 117:3605–3616. https://doi.org/10.1063/1.1495850.

55. Merzel, F., and J. C. Smith. 2002. Is the first hydration shell of lysozyme of higher density than bulk water? *Proc. Natl. Acad. Sci. USA.* 99:5378–5383. https://doi.org/10.1073/pnas.082335099.

56. Zivanov, J., T. Nakane, and S. H. W. Scheres. 2020. Estimation of high-order aberrations and anisotropic magnification from cryo-EM data sets in RELION-3.1. *IUCrJ.* 7:253–267. https://doi.org/10.1107/S2052252520000081. https://www.ncbi.nlm.nih.gov/pubmed/32148853.

57. Punjani, A., J. L. Rubinstein, …, M. A. Brubaker. 2017. cryoSPARC: algorithms for rapid unsupervised cryo-EM structure determination. *Nat. Methods.* 14:290–296. https://doi.org/10.1038/nmeth.4169. https://www.ncbi.nlm.nih.gov/pubmed/28165473.

58. Grant, T., and N. Grigorieff. 2015. Measuring the optimal exposure for single particle cryo-EM using a 2.6 A reconstruction of rotavirus VP6. *eLife.* 4:e06980. https://doi.org/10.7554/eLife.06980. https://www.ncbi.nlm.nih.gov/pubmed/26023829.

59. Zivanov, J., T. Nakane, and S. H. W. Scheres. 2019. A Bayesian approach to beam-induced motion correction in cryo-EM single-particle analysis. *IUCrJ.* 6:5–17. https://doi.org/10.1107/S205225251801463X. https://www.ncbi.nlm.nih.gov/pubmed/30713699.

60. Beckers, M., A. J. Jakobi, and C. Sachse. 2019. Thresholding of cryo-EM density maps by false discovery rate control. *IUCrJ.* 6:18–33. https://doi.org/10.1107/S2052252518014434. https://www.ncbi.nlm.nih.gov/pubmed/30713700.

61. Beckers, M., C. M. Palmer, and C. Sachse. 2020. Confidence maps: statistical inference of cryo-EM maps. *Acta Crystallogr. D Struct. Biol.* 76:332–339. https://doi.org/10.1107/S2059798320002995. https://www.ncbi.nlm.nih.gov/pubmed/32254057.

62. Goddard, T. D., C. C. Huang, …, T. E. Ferrin. 2018. UCSF ChimeraX: Meeting modern challenges in visualization and analysis. *Protein Sci.* 27:14–25. https://doi.org/10.1002/pro.3235. https://www.ncbi.nlm.nih.gov/pubmed/28710774.

63. Pettersen, E. F., T. D. Goddard, …, T. E. Ferrin. 2004. UCSF Chimera–a visualization system for exploratory research and analysis. *J. Comput. Chem.* 25:1605–1612. https://doi.org/10.1002/jcc.20084. https://www.ncbi.nlm.nih.gov/pubmed/15264254.

64. Nazarov, S., A. Myasnikov, and H. Stahlberg. 2024. Cryo-EM structure of mouse heavy-chain apoferritin. https://doi.org/10.2210/pdb8RQB/pdb.

65. Maki-Yonekura, S., K. Kawakami, …, K. Yonekura. 2023. Measurement of charges and chemical bonding in a cryo-EM structure. *Commun. Chem.* 6:98. https://doi.org/10.1038/s42004-023-00900-x. https://www.ncbi.nlm.nih.gov/pubmed/37258702.

66. Berman, H. M., J. Westbrook, …, P. E. Bourne. 2000. The Protein Data Bank. *Nucleic Acids Res.* 28:235–242. https://doi.org/10.1093/nar/28.1.235. https://www.ncbi.nlm.nih.gov/pubmed/10592235.

67. Kriegel, M., and Y. A. Muller. 2023. De novo prediction of explicit water molecule positions by a novel algorithm within the protein design software MUMBO. *Sci. Rep.* 13:16680. https://doi.org/10.1038/s41598-023-43659-w.

68. Rudling, A., A. Orro, and J. Carlsson. 2018. Prediction of Ordered Water Molecules in Protein Binding Sites from Molecular Dynamics Simulations: The Impact of Ligand Binding on Hydration Networks. *J. Chem. Inf. Model.* 58:350–361. https://doi.org/10.1021/acs.jcim.7b00520.

69. Biedermannová, L., and B. Schneider. 2015. Structure of the ordered hydration of amino acids in proteins: analysis of crystal structures. *Acta Crystallogr. D Biol. Crystallogr.* 71:2192–2202. https://doi.org/10.1107/S1399004715015679. https://www.ncbi.nlm.nih.gov/pubmed/26527137.

70. Nittinger, E., N. Schneider, …, M. Rarey. 2015. Evidence of water molecules–a statistical evaluation of water molecules based on electron density. *J. Chem. Inf. Model.* 55:771–783. https://doi.org/10.1021/ci500662d. https://www.ncbi.nlm.nih.gov/pubmed/25742501.

71. Pintilie, G., K. Zhang, …, W. Chiu. 2020. Measurement of atom resolvability in cryo-EM maps with Q-scores. *Nat. Methods.* 17:328–334. https://doi.org/10.1038/s41592-020-0731-1. https://www.ncbi.nlm.nih.gov/pubmed/32042190.

72. Berkeley, R. F., B. D. Cook, and M. A. Herzik, Jr. 2024. Machine learning approaches to cryoEM density modification differentially affect biomacromolecule and ligand density quality. *Front. Mol. Biosci.* 11:1404885. https://doi.org/10.3389/fmolb.2024.1404885. https://www.ncbi.nlm.nih.gov/pubmed/38698773.

73. Burley, S. K., H. M. Berman, …, C. Zardecki. 2022. Electron microscopy holdings of the Protein Data Bank: the impact of the resolution revolution, new validation tools, and implications for the future. *Biophys. Rev.* 14:1281–1301. https://doi.org/10.1007/s12551-022-01013-w. https://www.ncbi.nlm.nih.gov/pubmed/36474933.

Biophysical Journal, Volume *125*

Supplemental information

Predicting water at the protein interface in cryo-EM structures from MD-excess chemical potential

Qinfang Sun, Sriram Aiyer, Avik Biswas, Allan Haldane, Sompriya Chatterjee, Nobuyuki Matubayasi, Dmitry Lyumkis, and Ronald M. Levy

Supporting Material

Predicting Water at the Protein Interface in Cryo-EM Structures from MD-Excess Chemical Potential

Qinfang Sun[1,2], Sriram Aiyer[4], Avik Biswas[4,5], Allan Haldane[1,3], Sompriya Chatterjee[1,2,7], Nobuyuki Matubayasi[8], Dmitry Lyumkis[4,6*], Ronald M Levy[1,2,3*]

[1]Center for Biophysics and Computational Biology, Temple University, Philadelphia, PA, 19122;

[2]Department of Chemistry, Temple University, Philadelphia, PA, 19122;

[3]Department of Physics, Temple University, Philadelphia, PA, 19122;

[4]The Salk Institute for Biological Studies, La Jolla, CA, 92037;

[5]Department of Physics, University of California San Diego, La Jolla, CA, 92037;

[6]Graduate School of Biological Sciences, Department of Molecular Biology, University of California San Diego, La Jolla, CA, 92037;

[7]Material Science Institute, University of Oregon, Eugene, OR, 97403;

[8]Division of Chemical Engineering, Graduate School of Engineering Science, Osaka University, Toyonaka, Osaka 560-8531, Japan

*To whom correspondence should be addressed

*dlyumkis@salk.edu

*ronlevy@temple.edu

Table S1 Summary information of 9 cryo-EM structures of apoferritin (Group A) with resolution higher than 1.5 Å and 17 cryo-EM structures of apoferritin (Group B) with resolution between 1.5 and 2.0 Å.

PDB ID	Year of Deposition	Resolution (Å)	Refinement Software	Number of Cryo-EM Water per Chain
**Group A (9)**				
8RQB	2024	1.09	Unknown	110
7A6A	2020	1.15	Refmac	194
8J5A	2023	1.19	Refmac	145
7A4M	2020	1.22	Refmac	110
6Z6U	2020	1.25	Refmac	139
7RRP	2021	1.27	Phenix	102
7A6B	2020	1.33	Refmac	162
7K3V	2020	1.34	Phenix	111
7K3W	2020	1.36	Phenix	111
**Group B (17)**				
6Z9E	2020	1.55	Refmac	146
6Z9F	2020	1.56	Refmac	172
7R5O	2022	1.58	Refmac	139
7KOD	2020	1.65	Phenix	73
8EMQ	2022	1.66	Phenix	124
8EN7	2022	1.68	Phenix	106
6V21	2019	1.75	Phenix	75
8CPU	2023	1.76	Phenix	73
8CPV	2023	1.76	Phenix	71
8CPX	2023	1.76	Phenix	70
7ZG7	2022	1.77	Phenix	111
8CPT	2023	1.79	Phenix	79
8CPW	2023	1.79	Phenix	78
8CPM	2023	1.81	Phenix	81
8CPS	2023	1.82	Phenix	84
6S61	2019	1.84	Phenix	160
7VD8	2021	1.96	Phenix	103

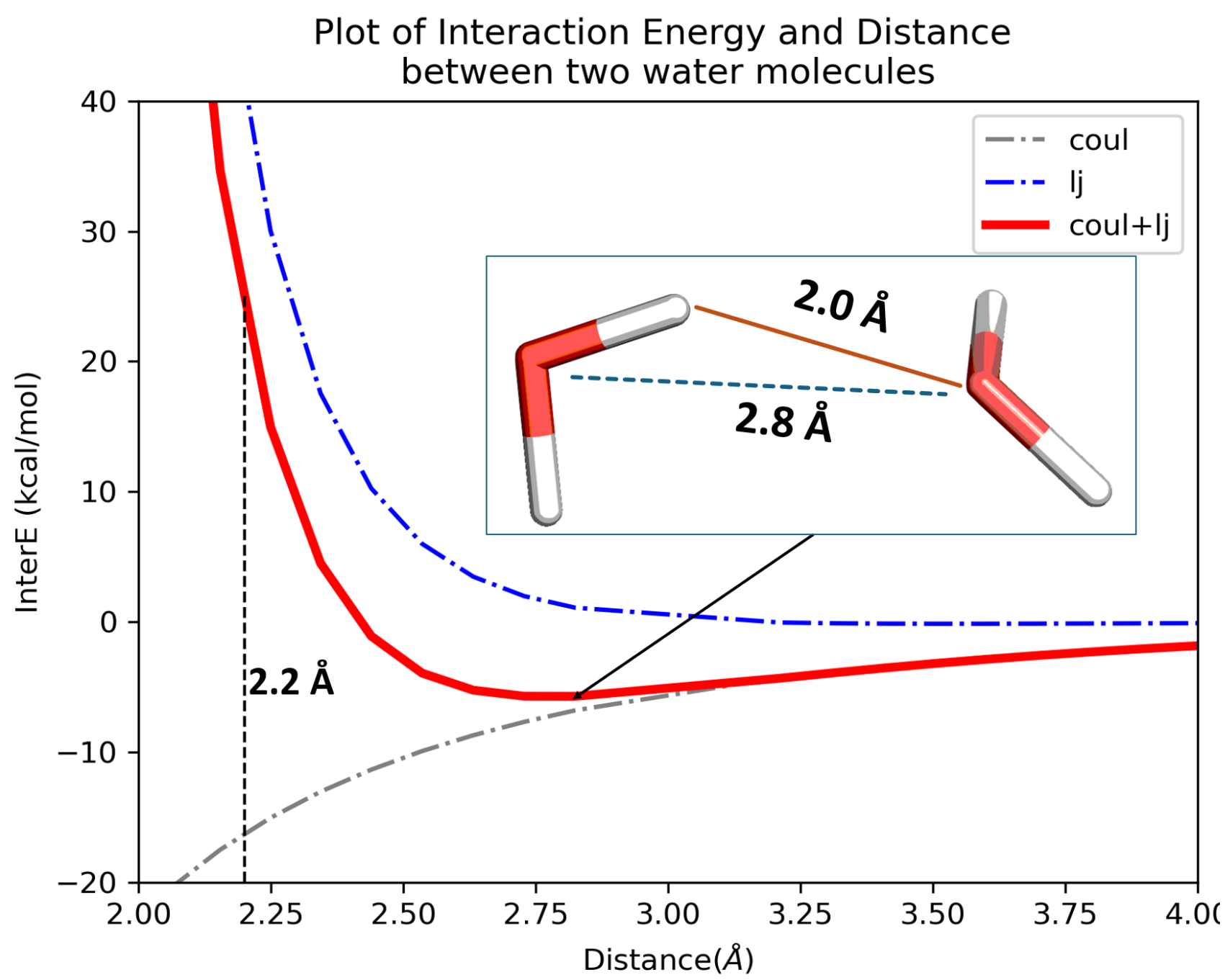

Figure S2: The interaction energy is plotted against the distance for two hydrogen-bonded water molecules (shown in inset) in gas phase using TIP3P model. The curves for Coulombic energy, Lennard-Jones energy and the total interaction energy are shown in grey, blue and red respectively.

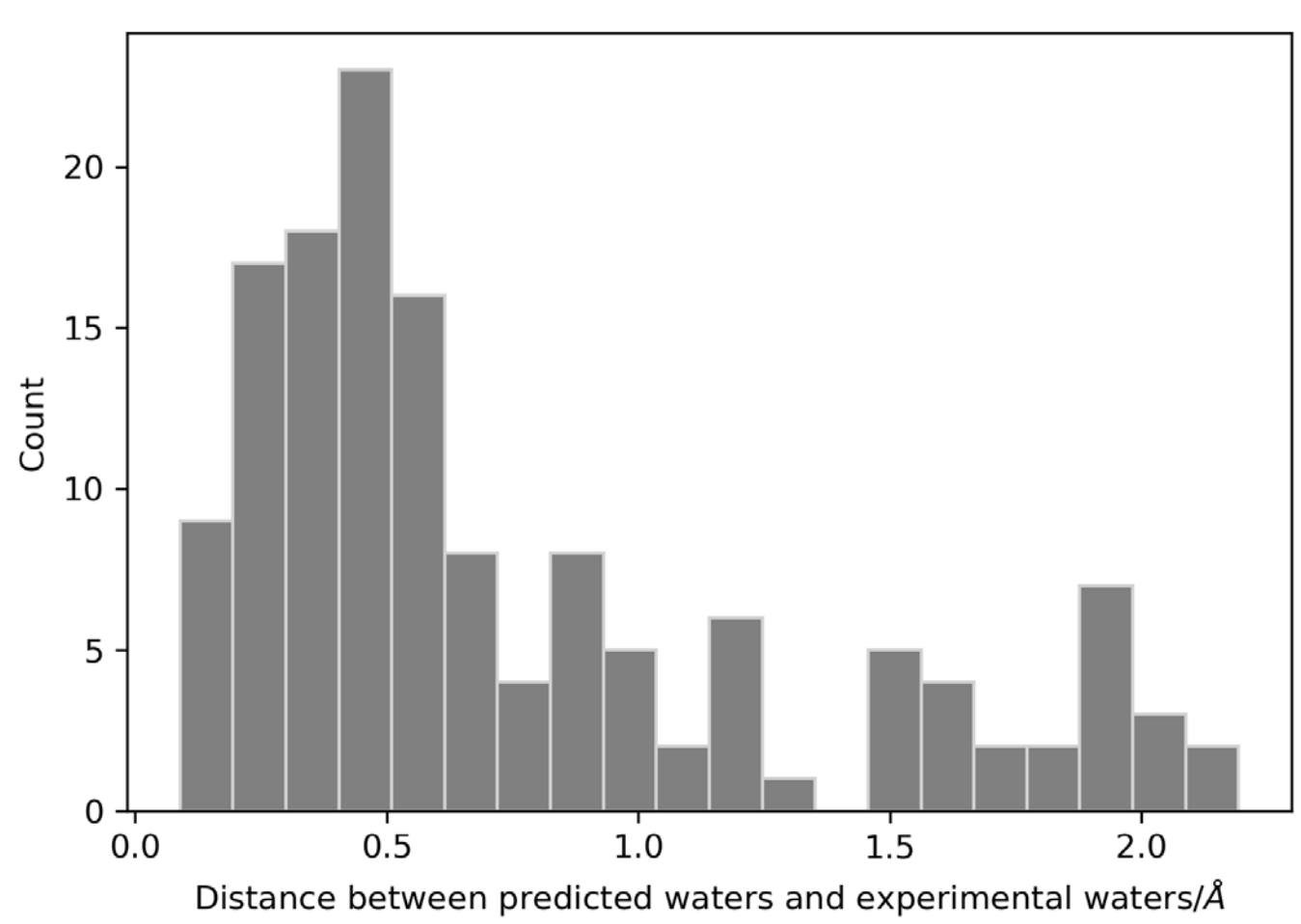

Figure S1: The histogram illustrates the distribution of distances between predicted waters and experimental waters with a 2.2 Å distance cutoff.

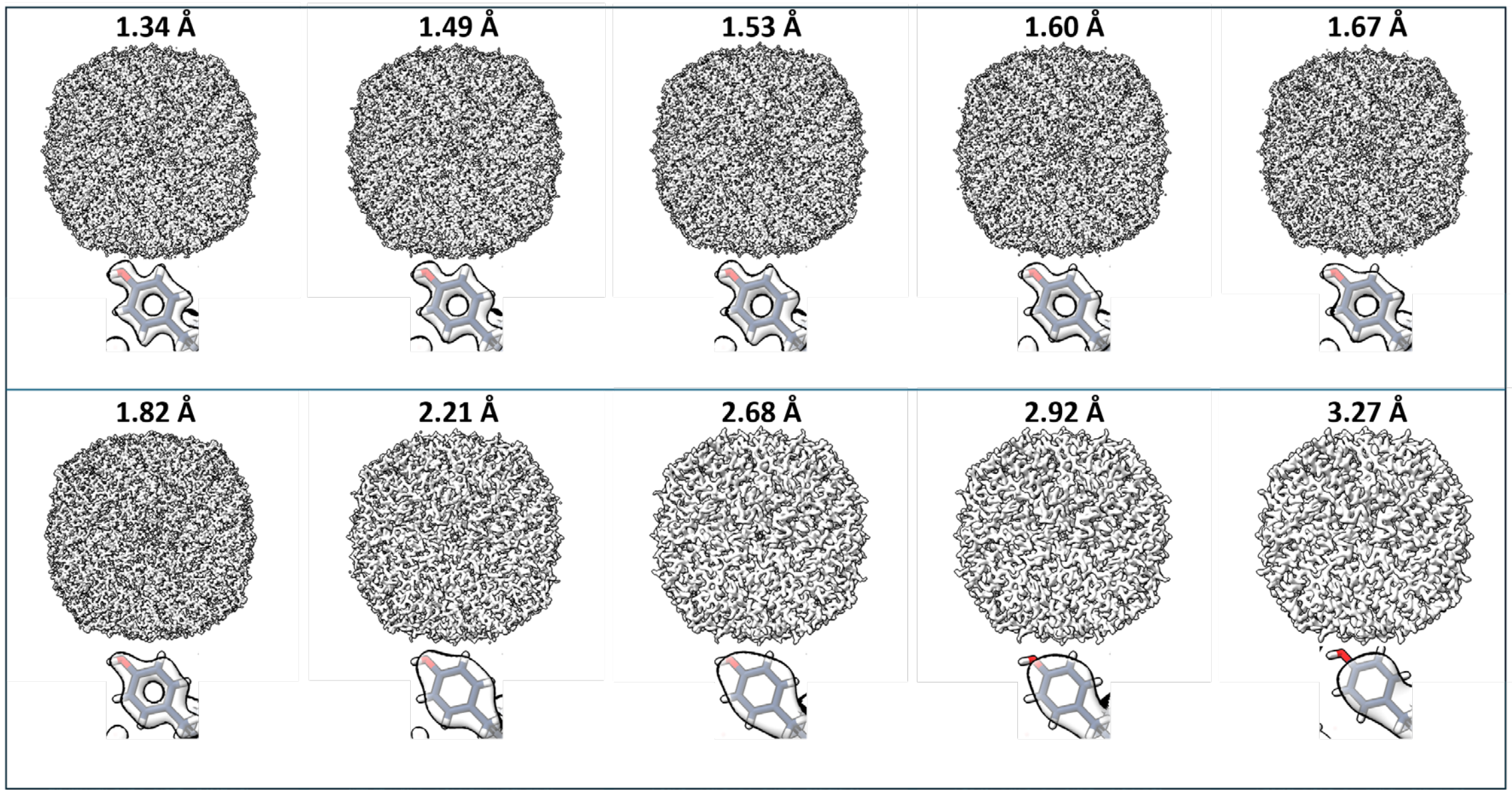

Figure S3: Final apoferritin confidence maps are shown at different resolutions (1.34 Å, 1.49 Å, ... 3.84 Å), with Y168 displayed at the bottom of each respective confidence map visualized at a 1% FDR threshold.

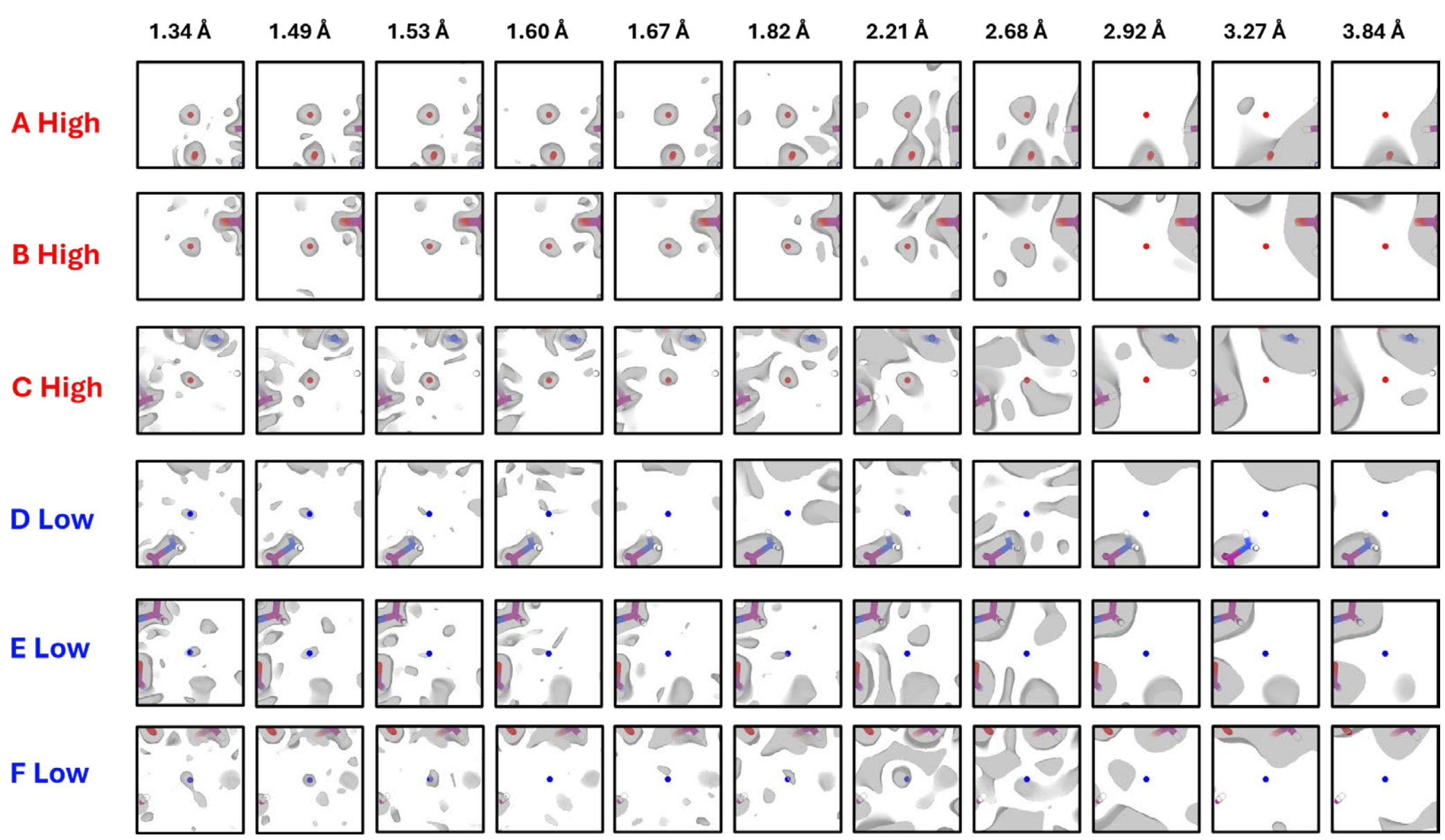

Figure S4: High-consensus (A–C) and low-consensus (D–F) waters are observed in confidence maps derived from cryo-EM data across a range of resolutions (1.34 Å, 1.49 Å, ... 3.84 Å) with a slightly expanded view that shows the surrounding residues. These maps are displayed at a 1% false discovery rate (FDR) threshold.

Table S2 The observation of high- and low-consensus waters in cryo-EM maps across various resolutions (1.34 Å, 1.49 Å, ... 3.84 Å) and at three different false discovery rate (FDR) thresholds: 0.1%, 1%, and 5%. At the different viewing thresholds, 'no' refers to no visible density, 'yes' refers to near-spherical density, 'partial' refers to near-spherical density with minor deviation or partial overlap from centroid position of water in the model, 'noisy' refers to density merging with density from neighboring atoms.

Water Confidence	Resolution (Å)									
	1.34	1.49	1.53	1.6	1.67	1.82	2.21	2.68	2.92	3.84
**lower**	No	No	No at 0.1%, partial overlap at 1 and 5%	No at 0.1 and 1%, partial at 5%	No at 0.1%, partial overlap at 1 and 5%	No	No	No	No	No
**lower**	No at 0.1 and 1%, partial overlap at 5%	No at 0.1%, partial overlap at 1 and 5%	No	No	No	No	Noisy and partial at 0.1%, noisy at 1 and 5%	Noisy and partial	No	No
**lower**	Yes	Partial	Partial at 0.1%, complet e at 1 and 5%	No at 0.1%, partial at 1%, complet e overlap at 5%	Partial	No at 0.1%, partial overlap at 1 and 5%	No	Partial at 0.1%, noisy at 1 and 5%	Noisy and partial at 0.1%, noisy at 1 and 5%	No
**lower**	No at 0.1%, partial at 1 and 5%	No at 0.1 and 1%, partial overlap at 5%	No	No at 0.1%, partial at 1 and 5%	No	No at 0.1%, partial overlap at 1 and 5%	No at 0.1 and 1%, partial overlap at 5%	No	No	No
**lower**	No	No	No	No	No	No	No	No	No	No
**lower**	Partial	Partial	No at 0.1%, partial overlap at 1 and 5%	No at 0.1%, partial at 1 and 5%	No at 0.1%, partial overlap at 1 and 5%	No at 0.1 and 1%, partial overlap at 5%	No at 0.1%, partial and noisy at 1 and 5%	No	No	No
**lower**	No at 0.1 and 1%, partial overlap at 5%	No at 0.1%, partial overlap at 1 and 5%	No	No at 0.1%, partial at 1 and 5%	No at 0.1 and 1%, partial overlap at 5%	Partial	No at 0.1%, partial overlap at 1 and 5%	No	No	No
**lower**	No at 0.1%, partial at 1 and 5%	No	No	No	No	No at 0.1%, partial overlap at 1 and 5%	Noisy and partial at 0.1%, noisy at 1 and 5%	Noisy and partial at 0.1 and 1%, noisy at 5%	No	No
**lower**	Partial	Partial	No at 0.1%, partial overlap at 1 and 5%	No at 0.1 and 1%, noisy at 5%	No	No at 0.1%, partial overlap at 1 and 5%	No	No	No	No
**lower**	Partial	Partial	No at 0.1 and	No at 0.1 and	No	No at 0.1%,	No	No	No	No

			1%, partial at 5%	1, noisy at 5%		partial overlap at 1 and 5%				
**lower**	Yes	Partial	Yes	Yes	Yes	Yes	Yes	Noisy	No at 0.1% and 1% and partial overlap at 5%	No
**lower**	Yes	Yes	Yes	Yes	Yes	Yes	Noisy	Noisy	No	No
**lower**	Yes	Yes	Yes	No at 0.1, noisy at 1 and 5%	Yes	Yes	Yes	No at 0.1, noisy and partial overlap at 1% and 5%	No	No
**lower**	Noisy	Noisy	Yes	Yes	Yes	Yes	Noisy	Noisy and partial	No	No
**lower**	Yes	Yes	Noisy	Yes	Yes	Partial at 0.1%, complet e at 1 and 5%	Yes	Yes	Yes	No
**lower**	Yes	Partial at 0.1 and 1%, complet e overlap at 5%	No at 0.1%, partial overlap at 1 and 5%	Yes	No at 0.1%, partial overlap at 1 and 5%	Partial	Yes	Partial at 0.1 and 1%, noisy at 5%	No	No
**lower**	Partial at 0.1%, complet e overlap at 1 and 5%	Yes	Partial at 0.1 and 1, complet e overlap at 5%	Partial at 0.1 and 1%, noisy at 5%	Partial at 0.1, complet e overlap at 1 and 5%	Yes	Yes	No at 0.1%, partial at 1 and 5%	No	No
**lower**	Partial at 0.1%, complet e overlap at 1 and 5%	Partial	Partial	No	No	No	No at 0.1%, partial and noisy at 1 and 5%	Noisy	No	No
**lower**	Yes	Yes and elongate d density	No at 0.1%, overlap at 1 and 5% with elongate d density	Yes	No at 0.1%, partial overlap at 1 and 5%	Partial	Noisy	Partial at 0.1 and 1%, partial and noisy at 5%	No	No
**lower**	Partial at 0.1 and 1%, complet e overlap at 5%	Yes	Partial at 0.1 and 1, complet e overlap at 5%	No	No	No at 0.1%, partial overlap at 1 and 5%	Noisy	Noisy	No	No
**lower**	Yes	Yes	Partial at 0.1, complet e overlap	Yes	Partial at 0.1, complet e overlap	Yes	Noisy	No	No	No

			at 1 and 5%		at 1 and 5%					
**lower**	Yes	Yes	Yes	Partial	Partial overlap at 0.1%, complete overlap 1 and 5%	Yes	Yes	No	No	No
**lower**	Yes	Partial at 0.1%, complete overlap at 1 and 5%	Yes	Partial at 0.1%, complete overlap at 1 and 5%	Partial	No at 0.1 and 1%, partial overlap at 5%	Noisy	Noisy	No	No
**lower**	Partial at 0.1%, noisy and partial at 1%, noisy at 5%	Yes	Yes	No	No	No at 0.1%, partial overlap at 1 and 5%	Noisy	No at 0.1%, noisy and partial at 1 and 5%	No	No
**high**	Yes	Yes	Yes	Yes	Yes	Yes	Yes	Noisy	No	No
**high**	Yes	Yes	Yes	Yes	Yes	Partial overlap at 0.1 and 1%, complete overlap at 5%	Noisy	Yes	No	No
**high**	Yes	Yes	Yes	Yes	Yes	Yes	Noisy	Yes	No	No
**high**	Yes	Yes	Yes	Yes	Yes	Yes	Yes	Noisy	Partial at 0.1%, noisy at 1 and 5%	No
**high**	Yes	Yes	Yes	Yes	Yes	Yes	Noisy	Partial at 0.1, noisy at 1 and 5%	No at 0.1%, noisy at 1 and 5%	No
**high**	Yes	Yes	Yes	Yes	Partial overlap at 0.1 and 1%, complete overlap at 5%	Partial overlap at 0.1 and 1%, complete overlap at 5%	Noisy	Noisy	No	No
**high**	Yes	Yes	Yes	Yes	Yes	Yes	Yes	Noisy	No at 0.1%, partial overlap at 1% and 5%	No
**high**	Yes	Yes	Yes	Yes	Partial at 0.1, complete overlap at 1 and 5%	Yes	Noisy	Partial at 0.1, complete overlap at 1, noisy at 5%	No	No

**high**	Yes	Yes	Yes	Yes	Yes	Yes	Yes	Partial at 0.1, complete overlap at 1 and 5%	No	No
**high**	Yes	Yes	Yes	Yes	Yes	Yes	Yes	Noisy	No at 0.1%, partial overlap at 1% and 5%	No
**high**	Yes	Yes	Yes	Yes	Yes	Yes	Yes	Noisy	No	No
**high**	Yes	Yes	Yes	Yes	Noisy	Noisy	No	No at 0.1, noisy and partial overlap at 1% and 5%	No at 0.1%, noisy at 1 and 5%	No
**high**	Yes	Yes	Yes	Yes	Yes	Yes	Yes	Noisy	No	No
**high**	Yes	Yes	Yes	Yes	Yes	Yes	Noisy	Noisy	Noisy	No
**high**	Noisy	Yes	Yes	Yes	Yes	Yes	Noisy	Noisy and partial at 0.1 and 1%, noisy at 5%	No	No
**high**	Yes	Yes	Yes	Yes	Yes	Yes	Yes	Partial at 0.1 and 1%, noisy at 5%	No	No
**high**	Yes	Yes	Yes	Yes	Yes	Yes	Noisy	Noisy	No	No
**high**	Yes	Yes	Yes	Yes	Yes	Yes	Noisy	Noisy and partial at 0.1 and 1%, noisy at 5%	No	No
**high**	Yes	Yes	Yes	Yes	Yes	Yes	Noisy	Partial at 0.1%, complete overlap at 1 and 5%	No at 0.1%, partial overlap at 1% and 5%	No
**high**	Yes	Yes	Yes	Yes	Yes	Yes	Noisy	Noisy	No	No
**high**	Yes	Yes	Yes	Yes	Yes	Yes	Noisy	Noisy	No at 0.1 and 1%, noisy at 5%	No
**high**	Yes	Yes	Yes	Yes	Yes	Yes	Yes	Yes	No	No
**high**	Yes	Yes	Yes	Yes	Yes	Yes	Yes	No at 0.1, noisy and partial overlap at 1% and 5%	No at 0.1, noisy and partial overlap at 1% and 5%	No

**high**	Yes	Yes	Yes	Yes	Yes	Yes	Noisy	Yes	No at 0.1%, partial at 1 and 5%	No
**high**	Yes	Yes	Yes	Yes	Yes	Yes	Noisy	Noisy	Noisy	No
**high**	Yes	Yes	Yes	Yes	Yes	Yes	Noisy	Yes	Partial at 0.1, complet e overlap at 1 and 5%	No
**high**	Yes	Yes	Yes	Yes	Yes	Yes	Yes	Yes	No at 0.1%, partial overlap at 1% and noisy at 5%	No
**high**	Yes	Yes	Yes	Yes	Yes	Yes	Noisy	Noisy	No at 0.1 and 1%, noisy at 5%	No
**high**	Yes	Yes	Yes	Yes	Yes	Yes	Noisy	Yes	Noisy	No
**high**	Yes	Yes	Yes	Yes	Yes	Yes	Noisy	Noisy	No	No
**high**	Yes	Yes	Yes	Yes	Yes	Yes	Noisy	Noisy	No at 0.1%, partial at 1 and 5%	No
**high**	Yes	Yes	Yes	Yes	Yes	Yes	Noisy	Noisy	No	No
**high**	Yes	Yes	Yes	Yes	Yes	Yes	Noisy	Noisy	No	No
**high**	Yes	Yes	Yes	Yes	Yes	Yes	Yes	Yes	No	No
**high**	Yes	Yes	Yes	Yes	Yes	Yes	Noisy	Noisy	No at 0.1% and 1%, noisy and partial overlap at 5%	No
**high**	Yes	Yes	Yes	Yes	Yes	Yes	Noisy	Yes	No	No
**high**	Yes	Yes	Yes	Yes	Yes	Yes	Yes	Partial	No	No
**high**	Yes	Yes	Yes	Yes	Yes	Yes	Yes	Noisy	No	No
**high**	Yes	Yes	Yes	Yes	Yes	Yes	Yes	No at 0.1, noisy and partial overlap at 1% and 5%	No	No
**high**	Yes	Yes	Yes	Yes	Yes	Yes	Noisy	Partial at 0.1%, noisy at 1 and 5%	No	No
**high**	Yes	Yes	Yes	Yes	Yes	Yes	Noisy	Noisy	No	No
**high**	Yes	Yes	Yes	Yes	Yes	Partial at 0.1 and	Yes	Noisy and	No	No

						1%, complete at 5%		partial at 0.1%, noisy at 1 and 5%		
**high**	Yes	Yes	Yes	Yes	Yes	Yes	Yes	Noisy	Yes	No
**high**	Yes	Yes	Yes	Yes	Yes	Noisy	Noisy	Noisy	No	No
**high**	Yes	Yes	Yes	Yes	Yes	Yes	Noisy	Noisy	No	No
**high**	Yes	Yes	Yes	Yes	Yes	Yes	Noisy	Yes	No	No
**high**	Yes	Yes	Yes	Yes	Yes	Partial at 0.1 and 1%, complete at 5%	Noisy	Yes	No	No
**high**	Yes	Yes	Yes	Yes	Yes	Yes	Noisy	Partial at 0.1%, complete overlap at 1 and 5%	No	No
**high**	Yes	Yes	Yes	Yes	Yes	Yes	Noisy	Noisy	Partial and noisy	No
**high**	Yes	Yes	Yes	Yes	Yes	Yes	Noisy	Partial at 0.1 and 1%, complete overlap at 5%	No	No
**high**	Yes	Yes	Yes	Yes	Yes	Yes	Yes	Noisy	No at 0.1%, partial at 1% and complete at 5%	No
**high**	Yes	Yes	Yes	Yes	Yes	Yes	Yes	Yes	No	No
**high**	Yes	Yes	Yes	Yes	Yes	Yes	Noisy	Yes	Noisy	No

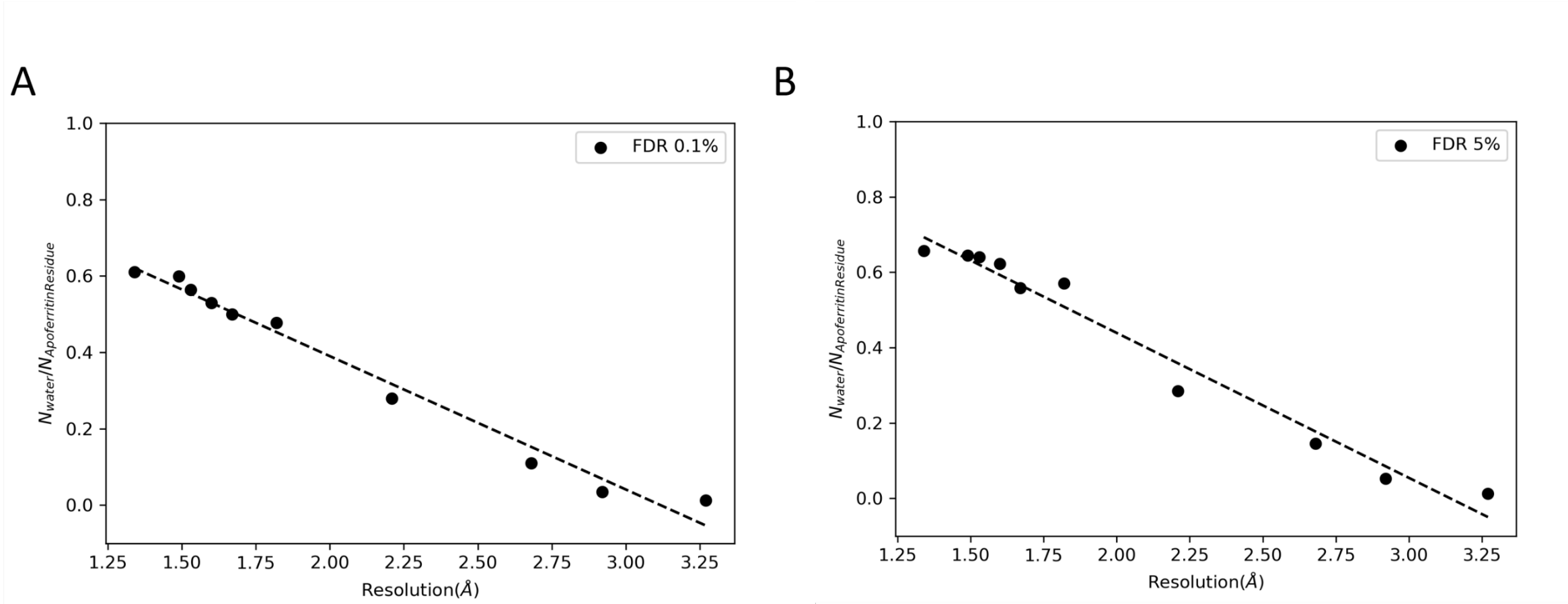

Figure S5: The plots showing the number of water molecules per apoferritin residue as a function of map resolution are based on data analyzed on confidence maps at 0.1% (**A**) and 5% (**B**) false discovery rate (FDR) thresholds.

Table S3 Comparison of water molecule rankings. (A) The top 60 water molecules ranked by their calculated interaction energies in Figure 3A. (B) The top 60 water molecules ranked by their WT values in Figure 3B. WT values were determined based on the experimentally resolved water positions. The interaction energy for each water molecule was computed with respect to the surrounding protein environment. Waters highlighted in green denote high-consensus sites, whereas waters highlighted in cyan indicate those that are present in both Table A and Table B.

A

WT_rank_ID	IE_rank_ID	cluster type	WT/kcal/mol	interEwo_Zn/kcal/mol	indirect part/kcal/mol
1	1	5	-4.72	-23.18	18.47
184	2	6	-1.01	-22.03	21.02
162	3	1	-2.67	-20.63	17.97
185	4	2	-1.00	-16.93	15.93
50	5	4	-4.19	-16.05	11.86
93	6	4	-3.65	-15.28	11.63
131	7	6	-3.13	-15.20	12.07
42	8	8	-4.27	-15.04	10.77
26	9	1	-4.43	-14.94	10.52

22	10	5	-4.44	-14.79	10.34
23	11	8	-4.44	-14.75	10.31
60	12	2	-4.05	-14.75	10.70
100	13	5	-3.51	-14.37	10.86
31	14	8	-4.38	-14.37	9.99
81	15	7	-3.82	-14.27	10.45
49	16	9	-4.19	-14.11	9.91
168	17	5	-2.53	-13.84	11.31
24	18	9	-4.44	-13.58	9.13
7	19	9	-4.60	-13.42	8.82
147	20	4	-2.92	-13.26	10.34
39	21	9	-4.31	-13.19	8.88
6	22	6	-4.61	-13.14	8.53
55	23	6	-4.15	-13.06	8.91
8	24	8	-4.59	-12.82	8.23
10	25	6	-4.57	-12.80	8.23
91	26	9	-3.69	-12.78	9.09
107	27	2	-3.43	-12.73	9.30
17	28	9	-4.51	-12.71	8.20
178	29	1	-1.67	-12.67	11.00
36	30	3	-4.35	-12.65	8.30
35	31	9	-4.35	-12.53	8.18
80	32	3	-3.82	-12.42	8.60
3	33	7	-4.66	-12.37	7.71
181	34	9	-1.46	-11.99	10.53
71	35	3	-3.93	-11.85	7.92
97	36	1	-3.54	-11.81	8.27

95	37	9	-3.62	-11.72	8.10
116	38	6	-3.34	-11.52	8.18
160	39	8	-2.67	-11.51	8.84
117	40	3	-3.33	-11.47	8.13
174	41	3	-2.20	-11.41	9.21
78	42	3	-3.85	-11.38	7.52
54	43	9	-4.16	-11.31	7.16
47	44	9	-4.20	-11.20	7.00
34	45	7	-4.35	-11.05	6.70
186	46	1	-1.00	-11.04	10.04
20	47	9	-4.49	-10.89	6.40
105	48	2	-3.47	-10.62	7.15
123	49	3	-3.24	-10.60	7.36
27	50	9	-4.41	-10.60	6.19
115	51	6	-3.34	-10.12	6.78
29	52	9	-4.39	-10.05	5.66
101	53	1	-3.51	-10.00	6.49
58	54	9	-4.09	-9.95	5.86
121	55	1	-3.25	-9.81	6.56
5	56	5	-4.62	-9.77	5.15
125	57	9	-3.19	-9.74	6.55
171	58	7	-2.43	-9.64	7.21
76	59	6	-3.87	-9.53	5.67
111	60	5	-3.40	-9.37	5.98
	ave	5.63 ± 2.85	-3.64 ± 0.96	-12.84 ± 2.78	9.2 ± 3.04

B

WT_rank_ID	IE_rank_ID	cluster type	WT/kcal/mol	interEwo_Zn/kcal/mol	indirect part/kcal/mol
1	1	5	-4.72	-23.18	18.47
2	141	9	-4.70	-5.35	0.66
3	33	7	-4.66	-12.37	7.71
4	71	6	-4.65	-8.36	3.71
5	56	5	-4.62	-9.77	5.15
6	22	6	-4.61	-13.14	8.53
7	19	9	-4.60	-13.42	8.82
8	24	8	-4.59	-12.82	8.23
9	86	7	-4.58	-7.30	2.71
10	25	6	-4.57	-12.80	8.23
11	104	8	-4.56	-6.62	2.06
12	91	9	-4.55	-7.16	2.61
13	132	8	-4.55	-5.64	1.09
14	153	7	-4.54	-4.84	0.30
15	62	8	-4.53	-9.32	4.79
16	168	9	-4.52	-3.94	-0.57
17	28	9	-4.51	-12.71	8.20
18	68	9	-4.51	-8.68	4.17
19	136	9	-4.50	-5.58	1.08
20	47	9	-4.49	-10.89	6.40
21	76	9	-4.46	-8.02	3.56
22	10	5	-4.44	-14.79	10.34

23	11	8	-4.44	-14.75	10.31
24	18	9	-4.44	-13.58	9.13
26	9	1	-4.43	-14.94	10.52
25	98	9	-4.43	-6.84	2.41
27	50	9	-4.41	-10.60	6.19
28	142	3	-4.40	-5.35	0.95
29	52	9	-4.39	-10.05	5.66
30	124	6	-4.38	-5.90	1.52
31	14	8	-4.38	-14.37	9.99
32	128	9	-4.36	-5.81	1.45
33	94	9	-4.36	-7.02	2.66
35	31	9	-4.35	-12.53	8.18
34	45	7	-4.35	-11.05	6.70
36	30	3	-4.35	-12.65	8.30
37	118	9	-4.32	-6.27	1.95
38	152	9	-4.32	-4.86	0.55
39	21	9	-4.31	-13.19	8.88
41	61	9	-4.28	-9.36	5.09
40	160	3	-4.28	-4.43	0.16
42	8	8	-4.27	-15.04	10.77
43	150	9	-4.24	-5.00	0.76
44	80	9	-4.23	-7.69	3.46
45	66	8	-4.22	-8.94	4.73
46	75	9	-4.20	-8.18	3.97
47	44	9	-4.20	-11.20	7.00
48	180	1	-4.20	-2.00	-2.20
49	16	9	-4.19	-14.11	9.91

50	5	4	-4.19	-16.05	11.86
51	121	8	-4.17	-6.23	2.06
52	138	9	-4.17	-5.50	1.33
53	99	8	-4.17	-6.81	2.65
54	43	9	-4.16	-11.31	7.16
55	23	6	-4.15	-13.06	8.91
56	100	8	-4.15	-6.75	2.60
57	147	9	-4.09	-5.03	0.94
58	54	9	-4.09	-9.95	5.86
59	107	9	-4.05	-6.50	2.45
60	12	2	-4.05	-14.75	10.70
	ave	7.46 ± 2.21	-4.37 ± 0.17	-9.57 ± 4.01	5.19 ± 3.99
